# Quantitative recovery of methoxy poly(ethylene glycol) and PEGylated nanosystems from complex biological matrices

**DOI:** 10.1039/d6na00559d

**Published:** 2026-07-27

**Authors:** Kevin Coutu, Cloé Dupré, Nicolas Gaudreault, Amatus Ngabonziza Sangwa, Nicolas Bertrand, Andrea A. Greschner, Marc A. Gauthier

**Affiliations:** a Institut National de la Recherche Scientifique (INRS), EMT Research Center 531 Boul. des Prairies Laval Quebec H7V 1B7 Canada marc.andre.gauthier@inrs.ca; b Axe Endocrinologie et Néphrologie, Centre de Recherche du Centre Hospitalier Universitaire (CHU) de Québec-Université Laval, Pavillon CHUL 2705 Boul. Laurier Quebec City Quebec G1V 4G2 Canada; c Faculty of Pharmacy, Laval University 1050 Ave. de la Médecine Quebec City Quebec G1V 4G2 Canada

## Abstract

This study systematically screened deproteinization conditions for the selective and quantitative recovery of methoxy poly(ethylene glycol) (mPEG) and model PEGylated nanomedicines, either intact or as mPEG components, from full human serum and full rat liver lysate. Although mPEG and all PEGylated nanosystems were *a priori* soluble under all the deproteinization conditions investigated, their recovery from serum (as measured by the barium–iodide assay and/or ^1^H NMR spectroscopy) varied considerably depending on the agent employed. Surprisingly, acetonitrile (with or without acidic pre-treatment) was found to be the only broadly applicable agent for the reliable, selective, and quantitative recovery of mPEG and PEGylated nanosystems from both serum and liver lysate. With very few exceptions, neither the molecular characteristics of mPEG nor the composition of the nanosystems significantly influenced recovery under these conditions.

## Abstract 2

This study screened deproteinization conditions for the selective and quantitative recovery of methoxy poly(ethylene glycol) and model PEGylated nanomedicines from full human serum and rat liver lysate.

## Graphical abstract

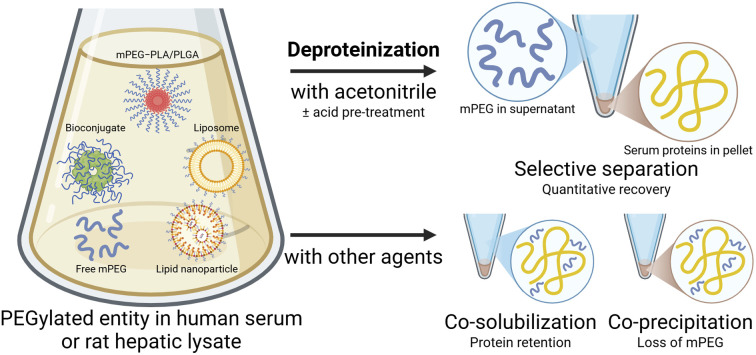

## Introduction

Methoxy poly(ethylene glycol) (mPEG) is widely used as a pharmaceutical excipient, and surface-grafting of mPEG to nanomedicines remains a central strategy to extend circulation time and reduce opsonization.^1–3^ The quantification of PEGylated nanosystems in serum is routinely performed to assess pharmacokinetics and biodistribution, typically using a fluorophore or radiolabel, and mPEG can even be quantified directly in serum by NMR spectroscopy.^4^ These techniques require minimal or no sample clean-up and thereby analyze the entire population of PEGylated species contained within. More complex evaluation of these systems by *e.g.*, chemical assays, chromatography, mass spectrometry, light scattering, *etc.*, generally requires removal of matrix molecules that might interfere with the analysis. Of course, for these analyses to be reliable, the clean-up procedure should recover the entire population of PEGylated species to avoid biases caused by partial recovery.^5,6^ It should also be known whether clean-up affects the chemical and physical integrity of the nanosystem to avoid biases when analyzing chemical degradation, disassembly, drug release, formation of coronas, *etc.*

Curiously, despite these considerations, the selectivity and yield of mPEG recovery as well as the stability of PEGylated nanosystems following common clean-up procedures, such as deproteinization,^7–10^ is rarely analyzed. Moreover, a systematic study that compares different PEGylated nanosystems and deproteinization conditions simply does not exist.

Deproteinization typically involves the aggregation, coalescence, and/or precipitation of proteins initiated by phenomena such as denaturation (acids, organic solvents), ion-pairing (hydrophobic salts), dehydration (salts, co-solvents), depletion interactions (molecular crowders), *etc.*^11,12^ As discussed above, deproteinization should ideally be achieved under conditions that do not lead to loss of mPEG in the precipitate and that minimize the co-recovery of serum components in the supernatant. Both outputs can be affected by mPEG–protein interactions before and during deproteinization. Within this context, our group has studied the complex equilibrium that exists between water, salts, mPEG, and proteins in solution and has shown that, depending on the concentrations of these four species, mPEG and protein interactions can be either dissociative or associative.^13,14^ Deproteinization agents will influence the amounts of available water/salts as well as the structure and surface chemistry of both proteins and PEGylated nanosystems. It is therefore reasonable to hypothesize that different deproteinization agents may either promote or disrupt mPEG–protein interactions, depending on how they specifically alter solution conditions. In addition, the intrinsic chemical and structural features of PEGylated nanosystems are already known to influence interactions with proteins (*e.g.*, formation of protein coronas),^15^ and can also be altered by deproteinization agents. Such effects could increase their propensity towards physical entrapment within or sticking to the surface of the protein pellet, or can chemically modify/destabilize the nanosystem, affecting how results are interpreted.

**Scheme 1:**
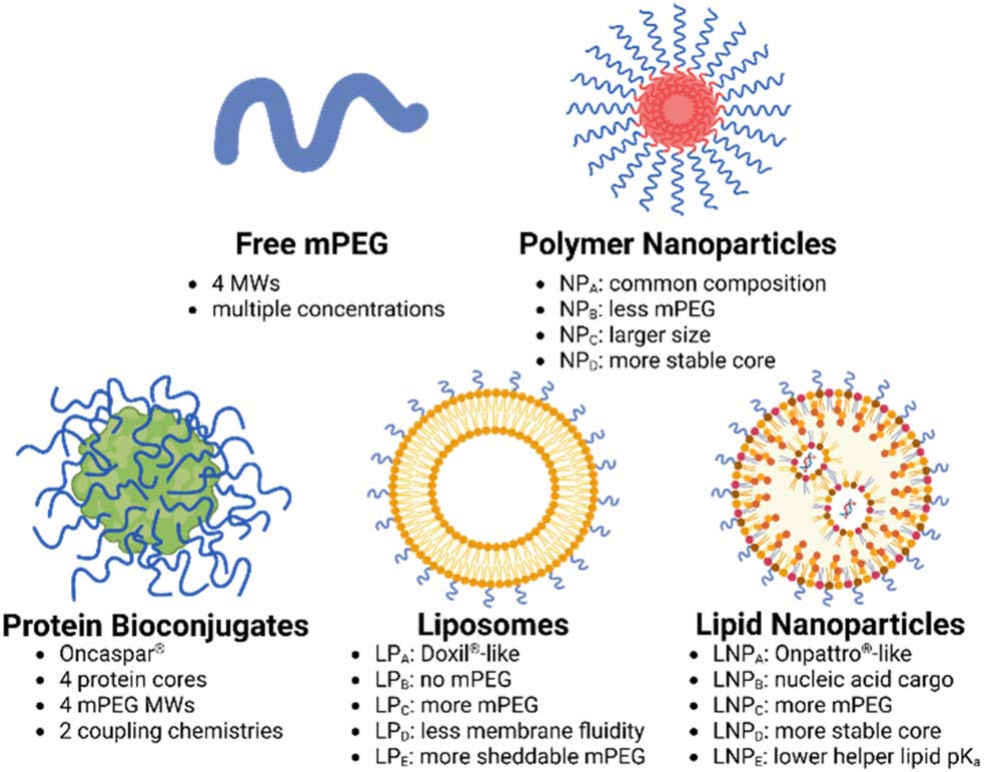
PEGylated nanosystems were prepared with features representative of those found in the literature, to evaluate their effect on recovery by deproteinization. Detailed characterization is provided in Tables S1–S4.

To explore these issues, several model nanosystems were prepared including PEGylated proteins (bioconjugates), polymeric NPs with poly(lactide-*co*-glycolide) cores, liposomes (LPs), and LNPs. For each system, key compositional and structural parameters were varied within ranges commonly reported in the literature (Tables S1–S4). Major features are summarized in Scheme 1. mPEG recovery was quantified either by the barium–iodide (BaI) assay^16^ and/or by proton nuclear magnetic resonance (^1^H NMR) spectroscopy (Scheme S1).^4,17^ Both methods have similar limits of detections, with the BaI assay having the advantage of being easily multiplexed for testing many samples, and the ^1^H NMR method having the advantage of being able to quantify mPEG in serum without deproteinization.^4^ The integrity of the nanosystems post deproteinization was analyzed by different techniques.

## Results and discussion

### Effects of deproteinization agents on mPEG recovery from full human serum

**Fig. 1:**
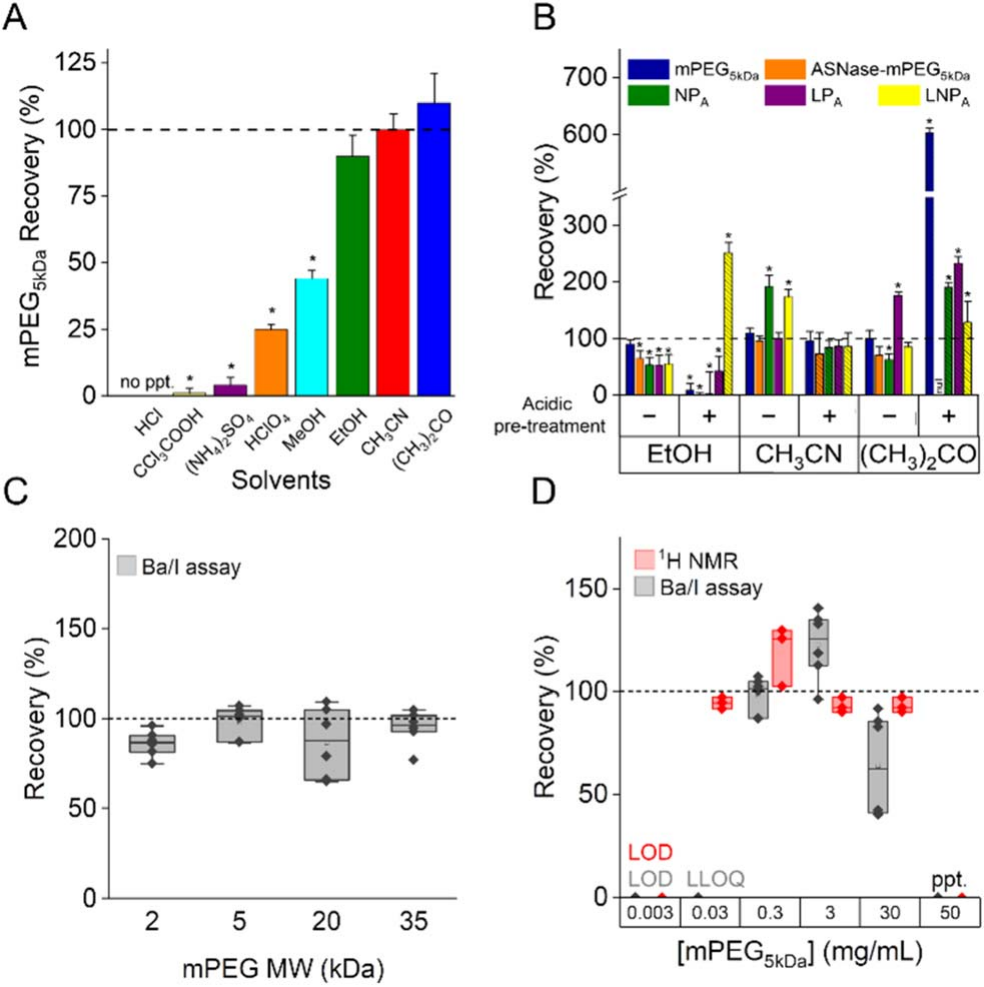
Screening deproteinization conditions for the recovery of mPEG and PEGylated nanosystems. (A) Broad screen showing that acetonitrile, acetone, and ethanol are the most promising deproteinization agents for recovery of mPEG_5kDa_. (Notes: HCl did not induce precipitation (no ppt.). Trichloroacetic acid interfered with the BaI assay, so ^1^H NMR spectroscopy was employed to determine recovery). (B) Refined screen for the PEGylated nanosystems. Hashed columns are for samples pre-treated with acid. (C and D) Recovery of mPEG was independent of MW and concentration. At 50 mg mL^−1^ mPEG-induced serum precipitation (ppt.) was observed. (A and B) Data are presented as mean ± s.d. (*n* = 1, in triplicate). (C and D) Box plots showing the interquartile range (25–75%, whiskers are 1.5 × s.d.), the median (solid line) and mean (square; *n* = 2 in triplicate). In all panes, “*” denotes a statistically significant difference from 100% recovery (ANOVA, Tukey, *p* < 0.05). Samples were 1 mg mL^−1^ unless otherwise stated. LOD: limit of detection; LLOQ: lower limit of quantification.

To begin elucidating the different possible effects of deproteinization agents, the recovery of free mPEG_5kDa_ was tested to gauge the intrinsic behavior of mPEG during deproteinization in pooled human serum (Fig. 1A). This was done before moving to more complex systems involving covalently modified mPEGs self-assembled into nanosystems (Fig. 1B). Several common deproteinization agents were tested: ammonium sulfate is a salting-out agent that induces protein precipitation by competition for water and by altering the surface tension of the solution; trichloroacetic acid and perchloric acid induce protein precipitation by changing the surface charge and folded structure of proteins through acidification and by increasing hydrophobicity through ion-pairing; the organic co-solvents methanol, ethanol, acetone, and acetonitrile cause protein precipitation by dehydration. Interestingly, pure aqueous solutions of mPEG_5kDa_ exposed to all these deproteinization agents remained homogenous, indicating solubility of mPEG_5kDa_ and, *a priori*, suitability for mPEG recovery from serum. However, as seen in Fig. 1A, deproteinization of full serum spiked with mPEG_5kDa_ resulted not only in protein precipitation but also in highly variable recovery of mPEG in the supernatant (1 ± 2%–110 ± 11%). This basic observation immediately illustrates that considerations beyond simple solubility of mPEG (and PEGylated nanosystems) in the deproteinization medium play a determining role in the selectivity and yield of mPEG recovery from serum. The recovery of mPEG_5kDa_ from serum using ammonium sulfate was very low (4 ± 3%). This is consistent with our previous observation that increased salt concentrations promote the co-association of mPEG and proteins in solution.^14^ Such a co-association could drag mPEG alongside the proteins into the precipitate either by physical entrapment or by sticking to the protein pellet. A similar loss of mPEG_5kDa_ from serum due to co-precipitation was observed using trichloroacetic acid (1 ± 2%) and perchloric acid (25 ± 2%). These agents mainly caused precipitation by increasing the hydrophobicity of proteins through ion pairing (HCl did not induce the precipitation of either serum proteins or mPEG_5kDa_). Finally, deproteinization using organic cosolvents substantially improved recovery from serum, indicating that these agents were better suited for preventing the loss of mPEG in the pellet. Nevertheless, mPEG recovery using methanol was moderate (44 ± 3%), whereas ethanol, acetonitrile, and acetone afforded quantitative recoveries of 90 ± 8%, 100 ± 6%, and 110 ± 11%, respectively.

In light of these results, the recovery of representative examples of each PEGylated nanosystem was analyzed using the three deproteinization agents affording quantitative recovery of mPEG_5kDa_ from serum (Fig. 1B). As for mPEG_5kDa_, aqueous solutions/dispersions of all the representative nanosystems remained macroscopically homogenous under deproteinization conditions. Interestingly, recovery from full serum changed based on the solvent used and on the type of nanosystem. Across all nanosystems, acetonitrile consistently afforded high recoveries, which were quantitative for ASNase–mPEG_5kDa_ (96 ± 9%; similar to Oncaspar®), and LP_A_ (100 ± 11%; similar to Doxil®), though exceeded 100% for NP_A_ and LNP_A_ (similar to Onpattro®). Surprisingly, ethanol generally yielded lower recoveries, mostly between ∼53–65%, while recovery using acetone was more variable (∼63–175%). These trends might be explained by the larger and denser precipitates formed during ethanol precipitation *vs.* acetonitrile/acetone, which could exacerbate physical entrapment of the nanosystems using ethanol (Fig. S1). The presence of the different nanosystems did not influence the morphology of the protein precipitate.

### Pre-treatment prior to deproteinization and stability of the nanosystems

Recovery values occasionally exceeding 100% in the BaI assay result from the co-recovery of serum molecules that happen to produce a signal in the BaI assay. Therefore, apparent recoveries above 100% are artificial and the result of an artefact specifically affecting the BaI assay. This artefact was observed consistently for the NP and LNP systems, but not for mPEG or the other PEGylated nanosystems tested (*vide infra*), suggesting formulation-dependent interactions with certain serum constituents leading to their co-recovery (note that the non-mPEG components of these nanosystems did not themselves produce a detectable signal in the BaI assay). While this artefact is inconsequential for mPEG quantification because other techniques such as NMR spectroscopy could be employed (*vide infra*), it represented an interesting case study for examining adaptations to acetonitrile deproteinization, to see whether they altered such co-recovery phenomena, and whether they affected the quantitative recovery of mPEG. More specifically, PEGylated nanosystem-spiked serum samples were subjected to basic or acidic pre-treatments prior to deproteinization. These pre-treatments were expected to alter any pH-dependent interactions between nanosystem and serum components and potentially hydrolyze nanosystem components involved in the co-recovery of residual serum constituents (many of the formulation components contain amphiphilic molecules with ester groups between their hydrophilic and hydrophobic moieties). Basic pre-treatment of NP_A_-spiked serum (1 M NaOH, pH ≈ 14, 1 h at 37 °C) followed by acetonitrile deproteinization yielded no measurable change in mPEG recovery (data not shown). In contrast, acidic pre-treatment (1 M HCl, pH ∼0.3, 1 h at 37 °C) prior to deproteinization resulted in quantitative values of mPEG recovery for all nanosystems using acetonitrile (Fig. 1B) by mitigation of the co-recovery of the interfering serum components. Curiously, acidic pre-treatment prior to ethanol or acetone deproteinization led to highly variable and irreproducible recovery of mPEG, sometimes yielding a total loss of mPEG, and sometimes exacerbating the recovery of serum components affecting the BaI assay (Fig. 1B). This result is consistent with the more variable recovery generally observed using these solvents, even in the absence of acidic pre-treatment (Fig. 1B). When analyzing residual absorbance at 280 nm and tryptophan fluorescence of the supernatants, no consistent trends were observed with respect to solvent, acidic pre-treatment, or the presence of mPEG and PEGylated nanosystems (Table S5 and Fig. S2). This lack of correlation between absorbance and fluorescence measurements suggests that the recovered species cannot be attributed solely to proteins. Rather, the observations are more consistent with the recovery of a heterogeneous mixture of serum molecules, whose composition depends on the solvent employed for deproteinization and specific interactions with the PEGylated nanosystems (if any).

From a physical point of view, no obvious differences in the morphology of the protein precipitates were observed due to the acidic pre-treatment (Fig. S1), except for ethanol for which pre-treatment yielded substantially finer precipitates (with the exception of NP_A_). DLS measurements suggest that the self-assembled mPEG-containing model NP_C_, LP_D_, and LNP_D_ nanosystems remain intact following acidic pre-treatment, though are disrupted upon addition of acetonitrile (Fig. S3). Furthermore, acidic pre-treatment did not induce detectable hydrolysis of mPEG, DSPE-mPEG_2kDa_, or DMG-mPEG_2kDa_ in any formulation as assessed by ^1^H NMR spectroscopy. Partial hydrolysis was observed only for the polyester component of NP_A_, suggesting a change of surface chemistry (Fig S4 and S5). Globally, these results suggest that the artefact observed for the NP and LNP systems is due to the solubilization of certain serum molecules by the dissolved components of the nanosystems. This solubilization appears to be pH-dependent, likely due to specific interactions with the formulation components, though the identity of the residual serum species was not investigated further.

Overall, acetonitrile was the only deproteinization agent that was broadly and reliably applicable across the four examples of PEGylated nanosystems tested for high mPEG recovery. However, NP_A_, LP_A_, and LNP_A_ were disrupted under these conditions and therefore their PEGylated components rather than the intact nanosystems themselves were recovered. Acidic pre-treatment could also be applied prior to deproteinization without detrimentally affecting the quantitative recovery of the PEGylated nanosystems or their components. These conditions were therefore carried forward to explore trends of mPEG molecular weight (MW) and PEGylated nanosystem composition.

### Effect of mPEG and PEGylated nanosystem characteristics

The influence of mPEG MW (2 to 35 kDa) and concentration (0.003–30 mg mL^−1^) are shown in Fig. 1C and D. Within these ranges, mPEG recovery was quantitative and independent of these variables (Fig. 1D). This independence suggests that recovery *via* acetonitrile deproteinization will remain relevant for clinically relevant samples, where circulating mPEG levels could be lower (0.01–6 µg mL^−1^).^18,19^

Given these promising results, bioconjugates were next examined. These are composed of a protein core and covalently attached mPEG chains whose relative abundance, surface accessibility, and spatial distribution can influence interactions with serum biomolecules and solubility during deproteinization. The bioconjugate panel included globular proteins spanning a wide range of sizes (20–345 kDa), charges, and isoelectric points (4.7–11.0): bovine serum albumin (BSA), *Escherichia coli*l-asparaginase (ASNase), human glutamate oxaloacetate transaminase (GOT), and hen egg-white lysozyme (HEWL). These proteins were modified with lysine-reactive mPEG (0.55–20 kDa) to generate nine bioconjugates with mPEG densities ranging from 0.9 to 22.7 mPEG chains per 100 nm^2^ of protein surface Table S1.

**Fig. 2:**
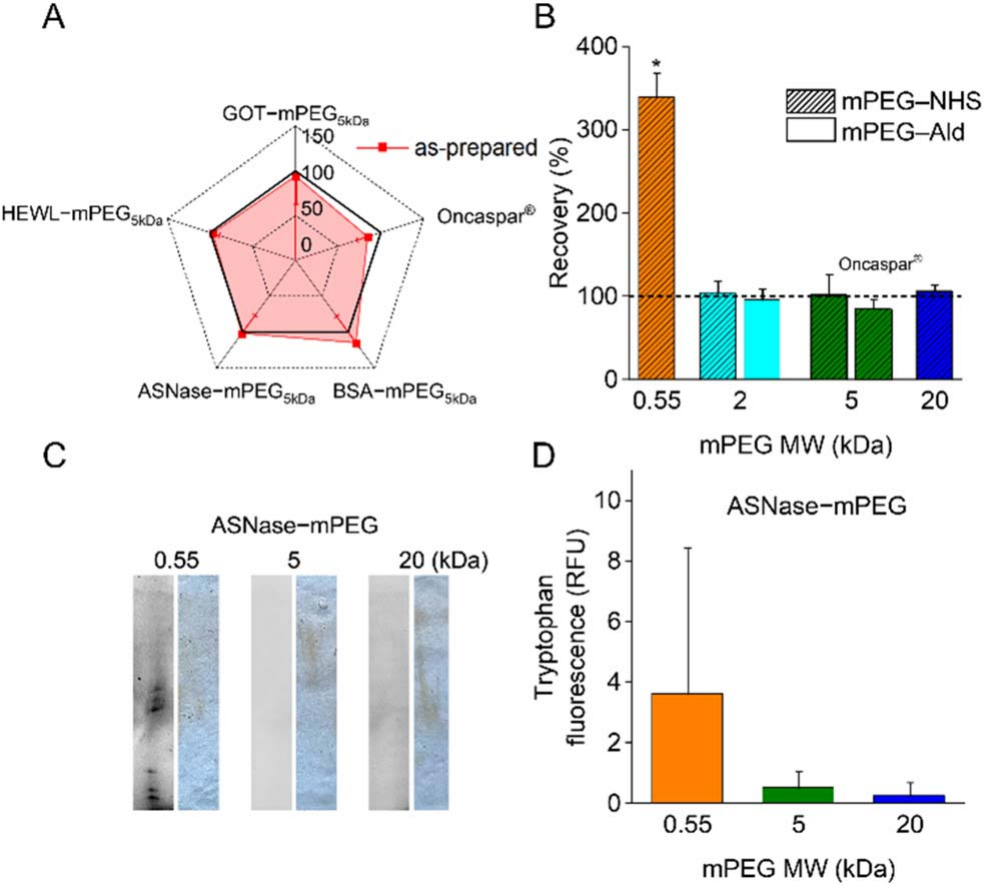
Recovery of PEGylated proteins from serum using acetonitrile, to explore the effects of bioconjugate structure. (A) The recovery of bioconjugates was independent of the core protein. (B) For the ASNase–mPEG bioconjugates, recovery was independent of mPEG MW, conjugation chemistry, and degree of PEGylation, except for mPEG_0.55kDa_, which was an outlier (see text). (C) SDS-PAGE stained for proteins (left) and for mPEG (right). (D) Tryptophan fluorescence indicates the presence of residual serum proteins recovered alongside ASNase–mPEG_0.55kDa_ during deproteinization. Data in Panes A, B, and D are presented as mean ± s.d. (*n* = 2, each in triplicate). Stars denote statistically significant difference from 100% recovery (ANOVA, Tukey, *p* < 0.05).

Initial testing focused on bioconjugates prepared with mPEG_5kDa_. As shown in Fig. 2A, acetonitrile deproteinization enabled quantitative recovery of all five bioconjugates, including Oncaspar®, independently of the identity of the core protein or mPEG grafting density. For ASNase bioconjugates prepared with mPEG_0.55–20kDa_ and two different coupling chemistries, recovery was likewise quantitative (Fig. 2B), except for ASNase–mPEG_0.55kDa_. For the latter, tryptophan fluorescence and SDS-PAGE, suggest the co-recovery of a small amount of serum proteins. No such co-recovery of proteins was observed with longer mPEG chains (Fig. 2C and D). SDS-PAGE further confirmed that the bioconjugates remained chemically intact following deproteinization, which is expected given that mPEG is covalently attached to the protein. Moreover, ^1^H NMR spectroscopy provided no evidence of bioconjugate degradation following acidic pre-treatment (Fig. S4). Rather, increased signal intensity in regions corresponding to hydrophobic and aromatic amino acid residues suggested changes in residue solvent accessibility (partial denaturation).

**Fig. 3:**
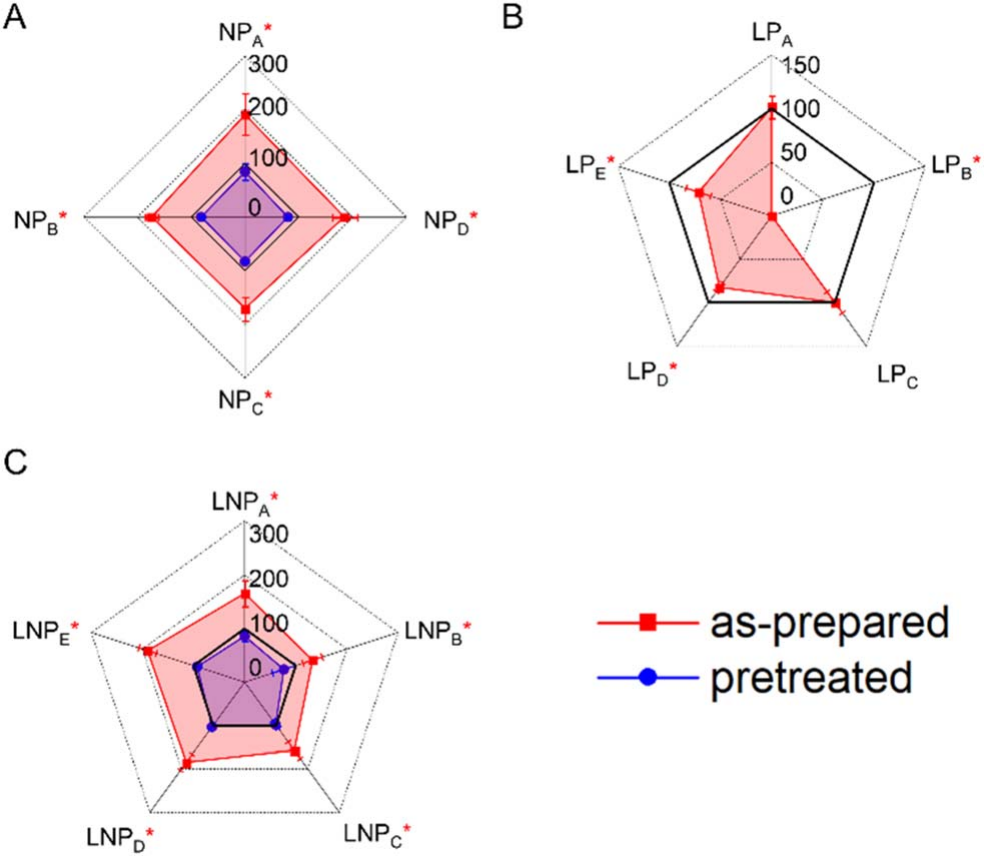
Recovery of PEGylated NPs, LPs, and LNPs from serum using acetonitrile, showing the effects of nanosystem composition and acidic pre-treatment. Using acetonitrile, the recovery of NPs (A) and LNPs (C) yielded values >100%, suggesting the co-recovery of serum biomolecules, while the recovery of PEGylated LPs (B) was quantitative (note: LP_B_ does not contain mPEG). Acidic pre-treatment of the PEGylated NPs and LNPs overcame this artefact, yielding quantitative mPEG recovery for all samples. Recovery was essentially independent of LP, NP, and LNP composition. Data presented as mean ± s.d. (*n* = 2, each in triplicate). Stars denote statistically significant difference from 100% recovery (ANOVA, Tukey, *p* < 0.05), and are color coded based on the figure legend.

PEGylated polymeric NPs were prepared by nanoprecipitation of PLA–mPEG with PLGA. The NPs possessed diameters of 98–180 nm and mPEG surface densities of 13–37 chains per 100 nm^2^ (Table S2). Hence, while larger than the bioconjugates, they possessed similar or higher mPEG coating densities. NP core composition, modulated by PLA–mPEG and PLGA, was varied to modulate NP size, stability, and mPEG surface density. All four PEGylated NPs (NP_A_–NP_D_) exhibited apparent recoveries from serum between ∼170–200%, with no statistically-significant differences between formulations (Fig. 3A). This result suggested the co-recovery of serum molecules as discussed above. Acidification of these samples prior to acetonitrile deproteinization overcame the co-recovery of serum contaminants affecting the BaI assay, yielding quantitative mPEG recovery (*i.e.*, ∼100%) for all NPs.

PEGylated LPs were prepared with mPEG chains bearing small lipid anchors (dimyristoyl glycerol (DMG) or 1,2-distearoyl-*sn*-glycero-3-phosphoethanolamine (DSPE)). The lipid anchor can influence membrane retention, with DMG-mPEG_2kDa_ being more likely to desorb from lipid assemblies in plasma or serum than DSPE-mPEG_2kDa_ due to the length of their alkyl chains (C_14_ and C_18_ for DMG and DSPE, respectively). A library of LPs was prepared with different mPEG surface densities (0–3.8 mPEG chains per 100 nm^2^) and spanning diameters ∼120–340 nm (Table S3). These formulations, LP_A_–LP_E_, were designed to probe the effects of mPEG density, lipid chemistry, and bilayer composition on recovery. As seen in Fig. 3B, recovery was quantitative for all LPs, with no apparent co-recovery of serum molecules affecting the BaI assay. These results indicate that the mPEG components of PEGylated liposomes (disrupted due to acetonitrile) can be quantitatively recovered from serum.

PEGylated LNPs contain ionizable lipids that are partially protonated at physiological pH. A library of LNPs, LNP_A_–LNP_E_, was prepared by varying ionizable lipid, helper lipid, cholesterol, and lipid-mPEG ratios to modulate mPEG surface density (19–42 mPEG chains per 100 nm^2^) and core stability (Table S4). As observed for the NPs, recovery of the LNPs by acetonitrile deproteinization reproducibly yielded values between ∼130–190% by the BaI assay across all formulations (Fig. 3C). Nevertheless, acidic pre-treatment mitigated the co-recovery of serum molecules affecting the BaI assay, yielding quantitative recoveries of the mPEG components of all the LNP formulations.

### Recovery following prolonged exposure to serum and liver lysate

**Fig. 4:**
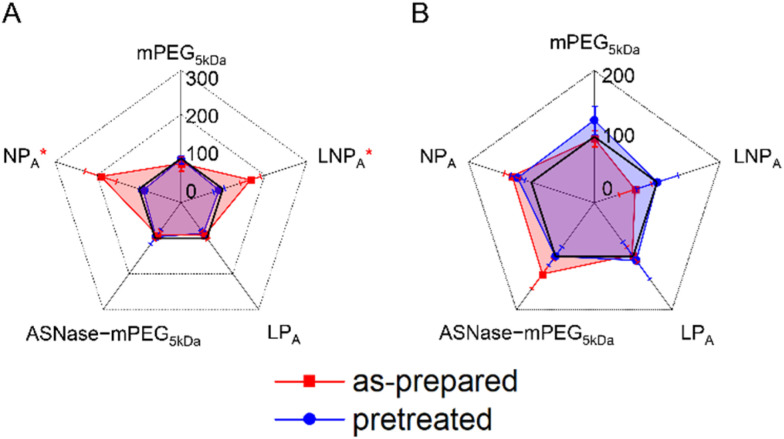
Recovery of PEGylated entities after prolonged exposure to serum and liver lysate. Following a 24 -h incubation in serum (A) and liver lysate (B), quantitative recovery could be achieved using acetonitrile either without, or with acidic pre-treatment to eliminate the co-recovery of contaminants. Note: In (B), ^1^H NMR spectroscopy was used to quantify mPEG (see text). Stars denote statistically significant difference from 100% recovery (ANOVA, Tukey, *p* < 0.05) and are color coded based on the figure legend.

Prolonged exposure of the PEGylated nanosystems to serum can favor the formation of an adsorbed ‘corona’ of biomolecules. This corona might, in turn, influence the behavior of the nanosystem during deproteinization. As such, a longer incubation time of 24 hours in full serum was tested, and Fig. 4A shows that this did not alter the recovery trends from preceding sections. That is, quantitative recovery of mPEG_5kDa_, ASNase–mPEG_5kDa_, and LP_A_ was achieved, and acidic pre-treatment did not affect these results. Likewise, deproteinization of NP_A_- and LNP_A_-spiked serum led to the co-recovery of serum molecules affecting the BaI assay, and this was also mitigated by acidic pre-treatment.

Finally, to gain a broader view of recovery of mPEG from other complex biological matrices, the PEGylated nanosystems were added to full rat liver lysate. Interestingly, in contrast to serum, the co-recovery of molecules from the liver lysate was observed for all PEGylated nanosystems, suggesting the recovery of molecules independent of specific interactions with the nanosystems, creating a very high background signal in the BaI assay (recoveries >200% systematically observed for all systems; Fig. S6). These contaminants could not be removed by acidic pre-treatment, suggesting that they are different from those in serum. Because of this, recovery was measured by ^1^H NMR spectroscopy, as these molecules from the liver lysate interfered much less with the mPEG-specific signal used for quantification (Fig. S7). As shown in Fig. 4B, the recovery of mPEG was quantitative for all PEGylated nanosystems, even after 24 -h exposure to the lysate. Recovery from full rat liver lysate was also unaffected by acidic pre-treatment, as measured by ^1^H NMR spectroscopy.

## Conclusions

This study shows that the recovery of mPEG and all PEGylated nanosystems from serum and liver lysate varied considerably depending on the deproteinization agent employed, due to loss in the protein pellet. Of the variety of conditions tested, this study concludes that the only widely applicable and reliable deproteinization agent for selective and quantitative recovery of mPEG and PEGylated nanosystems was acetonitrile. PEGylated bioconjugates were recovered intact in the supernatant, though the NPs, LPs, and LNPs were disrupted by acetonitrile and thus recovered as their components. Acidic treatment prior to acetonitrile deproteinization could equally be employed across all nanosystems, to modulate interactions with certain serum components (if desired) without compromising quantitative mPEG recovery. This treatment, however, partially denatured bioconjugates and partially hydrolyzed NPs. With very few exceptions, the molecular features of mPEG or the composition of the nanosystems did not influence results.

These results should be taken into consideration when analyzing results obtained for PEGylated nanosystems recovered from biological matrixes by deproteinization. When deproteinization induces biases that are incompatible with the sought after analysis, alternative clean-up procedures should be explored (*e.g.*, size-exclusion or affinity chromatography, immunoprecipitation, *etc.*).

## Conflicts of interest

The are no conflicts to declare.

## Supplementary Material

NA-008-D6NA00559D-s001

## Data Availability

The data supporting this article have been included as part of the supplementary information (SI). Supplementary information: experimental methods tabulated experimental data, supplementary results (recovery, DLS, NMR spectroscopy). See DOI: https://doi.org/10.1039/d6na00559d.

## References

[cit1] Veronese F. M., Pasut G. (2005). PEGylation, successful approach to drug delivery, Drug Discov. Today.

[cit2] Knop K., Hoogenboom R., Fischer D., Schubert U. S. (2010). Poly(ethylene glycol) in drug delivery: pros and cons as well as potential alternatives. Angew. Chem., Int. Ed..

[cit3] Shi D., Beasock D., Fessler A., Szebeni J., Ljubimova J. Y., Afonin K. A., Dobrovolskaia M. A. (2022). To PEGylate or not to PEGylate: immunological properties of nanomedicine's most popular component, polyethylene glycol and its alternatives. Adv. Drug Deliv. Rev..

[cit4] Alvares R. D., Hasabnis A., Prosser R. S., Macdonald P. M. (2016). Quantitative Detection of PEGylated Biomacromolecules in Biological Fluids by NMR. Anal. Chem..

[cit5] Hacene Y. C. (2021). *et al.*, Isolating Nanoparticles from Complex Biological Media by Immunoprecipitation. Nano Lett..

[cit6] Mui B. L. (2013). *et al.*, Influence of Polyethylene Glycol Lipid Desorption Rates on Pharmacokinetics and Pharmacodynamics of siRNA Lipid Nanoparticles. Mol. Ther. Nucleic Acids.

[cit7] Daramy K. (2024). *et al.*, Nanoparticle Isolation from Biological Media for Protein Corona Analysis: The Impact of Incubation and Recovery Protocols on Nanoparticle Properties. J. Pharm. Sci..

[cit8] Spinelli L. (2025). *et al.*, PEGylation-Driven Remodeling of the Protein Corona on PLGA Nanoparticles: Implications for Macrophage Recognition. Biomacromolecules.

[cit9] Caracciolo G., Farokhzad O. C., Mahmoudi M. (2017). Biological Identity of Nanoparticles In Vivo: Clinical Implications of the Protein Corona. Trends Biotechnol..

[cit10] The benefits and risks of PEGylation in nanomedicine, Nat. Nanotechnol., 2025, 20, 575, DOI: 10.1038/s41565-025-01951-y 40389642

[cit11] Alfano C. (2024). *et al.*, Molecular Crowding: The History and Development of a Scientific Paradigm. Chem. Rev..

[cit12] Zhou H. X., Rivas G., Minton A. P. (2008). Macromolecular Crowding and Confinement: Biochemical, Biophysical, and Potential Physiological Consequences. Annu. Rev. Biophys..

[cit13] Meziadi A., Bloquert V., Greschner A. A., de Haan H. W., Gauthier M. A. (2024). Harnessing Water Competition to Drive Enzyme Crosstalk. Biomacromolecules.

[cit14] Meziadi A., Zuberi N., de Haan H. W., Gauthier M. A. (2022). Overcoming PEG–Protein Mutual Repulsion to Improve the Efficiency of PEGylation. Biomacromolecules.

[cit15] Bertrand N. (2017). *et al.*, Mechanistic Understanding of *in Vivo* Protein Corona Formation on Polymeric Nanoparticles and Impact on Pharmacokinetics. Nat. Commun..

[cit16] Coutu K. (2025). *et al.*, Direct Quantification of PEGylation for Intact Bioconjugates and Nanoparticles by the Colorimetric Barium/Iodide Assay. Biomacromolecules.

[cit17] Zaghmi A. (2019). *et al.*, Determination of the Degree of PEGylation of Protein Bioconjugates Using Data from Proton Nuclear Magnetic Resonance Spectroscopy. Data in Brief.

[cit18] Fishburn C. S. (2008). The Pharmacology of PEGylation: Balancing PD with PK to Generate Novel Therapeutics. J. Pharm. Sci..

[cit19] Baumann A., Tuerck D., Prabhu S., Dickmann L., Sims J. (2014). Pharmacokinetics, Metabolism and Distribution of PEGs and PEGylated Proteins: Quo Vadis?. Drug Discov. Today.

