# Supplementary material for: Quantitative recovery of methoxy poly(ethylene glycol) and PEGylated nanosystems from complex biological matrices
Source: Nanoscale Adv. 2026 Jul 27;8(16):4672–7. doi: 10.1039/d6na00559d (PMC13404359; doi:10.1039/d6na00559d)
Supplement: Supplementary file 1 [file NA-008-D6NA00559D-s001.pdf]

Supplementary Information (SI) for Nanoscale Advances.
This journal is © The Royal Society of Chemistry 2026

Supporting information

for

Quantitative recovery of methoxy poly(ethylene glycol) and PEGylated nanosystems from complex biological matrices

Kevin Coutu[a], Cloé Dupré[a], Nicolas Gaudreault[b,c], Amatus Ngabonziza Sangwa[a], Nicolas Bertrand[c,b], Andrea A Greschner[a], and Marc A Gauthier[a,*]

[a] Institut National de la Recherche Scientifique (INRS), EMT Research Center, 531 boul. des Prairies, Laval, Quebec, H7V 1B7, Canada

[b] Axe Endocrinologie et Néphrologie, Centre de recherche du Centre Hospitalier Universitaire (CHU) de Québec – Université Laval, Pavillon CHUL, 2705 boul. Laurier, Quebec City, Quebec, G1V 4G2, Canada.

[c] Faculty of Pharmacy, Laval University, 1050 ave. de la Médecine, Quebec City, Quebec, G1V 4G2, Canada

Experimental Section

1.1 Materials and Reagents. All chemicals were of analytical grade and were used as received. Amicon Ultra centrifugal filters (30 and 50 kDa molecular weight cut-off, MWCO), acetone (CH_3COCH_3), acetonitrile (ACN, CH_3CN), ammonium sulfate ($(NH_4)_2SO_4$), deuterated chloroform ($CDCl_3$), cholesterol (Chol.), hen egg-white lysozyme (HEWL), iodine (I_2), 1,5,7-triazabicyclo[4.4.0.]dec-5-ene (TBD), poly(adenosine) (poly(A)), perchloric acid ($HClO_4$), mPEG−OH (2–35 kDa), phosphate buffered saline (PBS, 10 mM phosphate, 137 mM NaCl, pH 7.4), and trichloroacetic acid (TCA, CCl_3COOH) were obtained from Sigma-Aldrich (Oakville, ON, Canada). Lipids, including 1,2-dioleoyl-*sn*-glycero-3-phosphoethanolamine (DOPE), 1,2-dioleoyl-3-dimethylammonium-propane (DODAP), 1,2-distearoyl-*sn*-glycero-3-phosphocholine (DSPC), and 1,2-dimyristoyl-*sn*-glycero-3-phosphoethanolamine-*N*-$mPEG_{2kDa}$ (DMG−$mPEG_{2kDa}$), and 1,2-distearoyl-*sn*-glycero-3-phosphoethanolamine-*N*-($mPEG_{2kDa}$) (DSPE–$mPEG_{2kDa}$) were purchased from NOF America Corporation (Boston, MA). The ionizable lipid heptatriaconta-6,9,28,31-tetraen-19-yl 4-(dimethylamino)butanoate (MC3) was obtained from Cayman Chemical (Ann Arbor, MI). 4-(2-Hydroxyethyl)piperazine-1-ethanesulfonic acid (HEPES), sodium citrate, and sodium chloride were obtained from Fisher Scientific (Ottawa, Canada). Barium chloride ($BaCl_2$), bovine serum albumin (BSA, fraction V), hydrochloric acid (HCl), and sodium hydroxide (NaOH) were from BioShop (Burlington, ON). Citric acid was from Laboratoire MAT (Québec, Canada). mPEG aldehyde (mPEG−Ald), and mPEG *N*-hydroxysuccinimide ester (mPEG−NHS) with molecular weights between 0.55−20 kDa were purchased from Creative PEGWorks (NC, USA) and Thermo Fisher Scientific (Saint-Laurent, QC, Canada). Additional proteins tested included human glutamate oxaloacetate transaminase (GOT) (Lee Biosolutions, MO, USA), and *E. coli* L-asparaginase (ASNase; Xiamen Hisunny Chemical Co., China). Oncaspar® (pegaspargase) was kindly provided by the CHU de Québec-Université Laval and purified by centrifugal dialysis (30 kDa MWCO) against 100 mM phosphate buffer (pH 8.0) to remove formulation excipients. Poly(D/L-lactide-*co*-glycolide) (50:50, 30 kDa) ($PLGA_{30kDa}$, Lactel®, B6013-2P) was purchased from Durect Corporation (Birmingham, USA). Poly(D/L-lactide-*co*-glycolide) (50:50, 95 kDa) ($PLGA_{95kDa}$, Purasorb®, PDLG5010) was purchased from PURAC Biochem (Gorinchem, The Netherlands). Poly(lactic acid)–methoxy poly(ethylene glycol) (PLA_{25kDa}–$mPEG_{5kDa}$) was synthesized according to a previously reported procedure.[1] Protein extinction coefficients at 280 nm (M^{-1} cm^{-1}), calculated from the amino acid sequence and used for protein quantification, were as follow: ASNase and Oncaspar® 23,630; GOT 68,005; BSA 52,165; and HEWL 38,400.

1.2 PEGylated proteins. Select mPEG−protein conjugates (ASNase, GOT, BSA, and HEWL) were prepared in our previous report and used without further modification.[1] The degree of PEGylation was determined by ^{1}H NMR spectroscopy following the reported method,[2] in which lyophilized bioconjugates were dissolved in D_2O and the mPEG methylene signal at 3.6 ppm was quantified relative to an internal DMSO standard. Detailed compositions and physicochemical characterizations of these conjugates are provided in **Table S1**.

1.3 PEGylated polymer nanoparticles (NPs). mPEG−PLA copolymers were synthesized via ring-opening polymerization of lactide as described previously.[3] The resulting polymers were used without further modification. NPs composed of combinations of $mPEG_{5kDa}$−PLA_{25kDa}, $PLGA_{30kDa}$ and $PLGA_{95kDa}$ were obtained via nanoprecipitation from acetonitrile into water following our previously reported method.[3] As-prepared NP solutions were lyophilized, and the dry polymers were dissolved in $CDCl_3$ to determine the mPEG mass fraction by NMR spectroscopy. The relative integrations of the mPEG methylene (3.6 ppm), lactide (4.7 ppm), and glycolide (5.2 ppm) resonances were used, considering their respective molecular weights, assuming that mPEG chains are fully solvent-exposed. For convenience, detailed compositions and physicochemical characterizations of these formulations are provided in **Table S2**.

1.4 PEGylated liposomes (LPs). Five liposomal formulations were prepared by thin-film hydration followed by extrusion. Lipids (DSPC, DOPE, Chol., DSPE−$mPEG_{2kDa}$, and DMG−$mPEG_{2kDa}$) were first dissolved in ethanol at a concentration of 10 mg mL^{-1} and mixed in a 10 mL round-bottom flask to obtain formulations with the compositions detailed in **Table S3**. The mPEG content in the formulations was calculated from the initial molar fraction of lipid–mPEGs (DSPE–$mPEG_{2kDa}$ and DMG–$mPEG_{2kDa}$) in the lipid mixture. Ethanol was evaporated under vacuum using a rotary evaporator at 50 °C to form a homogeneous lipid film. The films were hydrated with 0.8 mL of HEPES-buffered saline (HBS, 10 mM, pH 7.4) and incubated at 50 °C for 90 min. The hydrated films were briefly sonicated and vortexed to ensure complete hydration. The liposomal suspensions were then extruded through polycarbonate membranes with pore sizes of 200, 100, and 50 nm using a manual extruder (Avestin Inc., Ottawa, ON, Canada). Cholesterol and phosphate contents were determined using the Cholesterol E assay (Fujifilm).[4] and a modified Bartlett assay.[5]

1.5 PEGylated lipid nanoparticles (LNPs). Lipids (DSPC, DOPE, Chol, the ionizable lipids DODAP or MC3, and the lipid–mPEGs DMG–$mPEG_{2kDa}$ or DSPE–$mPEG_{2kDa}$) were first dissolved in ethanol at a concentration of 10 mg mL^{-1} and mixed to yield the desired formulations (**Table**

S4). The mPEG content in the LNPs was calculated from the initial molar fraction of DMG–$mPEG_{2kDa}$. The ethanolic suspensions were then diluted to obtain a total lipid concentration of 10 mM. LNPs were prepared by rapid mixing of the ethanolic and aqueous phases using a T-junction and two syringe pumps (New Era Instruments, NE-1000) at a total flow rate of 4 mL min^{-1} with a 1:3 volume ratio. The aqueous phase consisted of 10 mM citrate buffer (pH 4.0). In formulation LNP_B, poly(A) was added as a nucleic acid cargo at a weight ratio of 10:1 (MC3:poly(A)). After mixing, LNPs were diluted threefold with PBS and briefly vortexed. Residual ethanol and citrate were removed by dialysis (Slide-A-Lyzer, MWCO 10 kDa; Thermo Fisher Scientific) against PBS (500× volume) for 5 h at ambient temperature. LNPs were then concentrated using ultrafiltration units (Amicon Ultra-15, MWCO 100 kDa), filtered through a 0.22 µm syringe filter, and stored at 4 °C until use. Cholesterol content in the formulations was determined using the Cholesterol E assay.

1.6 Size analysis. The size (Z-average) and polydispersity index (PDI) were determined by dynamic light scattering (DLS; NanoSizer S, Malvern Panalytical, Malvern, UK) at a scattering angle of 173º with a 633 nm laser. Samples were diluted 100-fold before analysis (H_2O for NPs, HEPES-buffered saline pH 7.4 for LPs, and PBS for LNPs).

1.7 Spiked serum and deproteinization. Commercial pooled human AB serum (Human AB Serum, sterile; Corning, Mediatech, Inc., Manassas, VA, USA; Cat. No. 35-060-CI) was stored at 4 ºC and aliquoted aseptically immediately before use. Stock solutions of mPEG and of each PEGylated entity were first prepared in PBS at known concentrations. These stock solutions were serially diluted as needed and analyzed by the barium–iodide (BaI) assay (*vide infra*) to validate their mPEG content and to confirm the linear range of quantification for each species. For all experiments, the objective was to spike serum with a fixed volume of stock solution to achieve target mPEG concentrations in the range of approximately 0.03 to 30 mg mL^{-1}. To ensure consistent handling across experiments, the volume of stock solution added to serum was kept within a narrow range (1–2 µL), resulting in samples containing more than 95% serum. When lower amounts of mPEG were required, stock solutions were diluted in PBS prior to addition to serum, thereby maintaining comparable serum dilution across conditions. PEGylated nanomedicines were treated similarly. Spiked serum samples were handled at room temperature for less than 5 minutes before deproteinization or incubated aseptically for up to 24 h before deproteinization to exacerbate possible interactions with serum biomolecules.

Deproteinization was carried out by adding three volume equivalents of the deproteinization agent at room temperature (150 µL of deproteinization agent for 50µL volume of serum). The agents

evaluated included aqueous solutions of 1 M HCl, 1 M $HClO_4$, and 1 M CCl_3COOH, as well as pure organic solvents (MeOH, EtOH, CH_3CN, and $(CH_3)_2CO$; >99%). Samples were vortexed immediately and centrifuged at 14,000 × g for 5 minutes at 4 ºC. Supernatants were collected and analyzed by the BaI assay or by NMR spectroscopy. Some samples were subjected to acidic pre-treatment. For this pre-treatment, 50 µL spiked serum samples were incubated with one volume equivalent (50 µL) of 1 M HCl at 37 ºC for 1 hour. Thereafter, deproteinization was carried out by addition of three volume equivalents (300 µL) of acetonitrile, followed by vortexing, centrifugation, and supernatant collection. For each extraction condition, negative controls (PBS-spiked serum) and positive controls (water replacing serum) were included to account for matrix effects.

1.8 BaI assay. Supernatants from above were processed as follows. Ten-microliter (10 µL) aliquots were diluted with 300 µL of Milli-Q water, followed by the addition of 120 µL of barium chloride solution (5 wt% in 1 M HCl). Subsequently, 130 µL of each mixture were transferred in triplicate to a 96-well quartz microplate, and 20 µL of iodine/iodide solution (10 mM I_2, 2 wt% NaI in water) were dispensed into these wells using a multichannel pipette. Absorbance was measured at 535 nm within 5 min of iodine/iodide addition. Blanks consisted of distilled water in place of the sample. Additional wells containing equivalent volumes of pure Milli-Q water were measured to manually correct for pathlength by normalizing absorbance values to the mean signal of these water wells rather than using the instrument's built-in pathlength correction function. Automatic pathlength correction was not suitable because it relies on water absorbance measurements at 977 and 900 nm, wavelengths at which the BaI assay also produces a measurable signal.

$$\textit{Equation 1: Pathlength} = (A_{977nm} - A_{900nm}) / 0.182\ \text{cm}^{-1}$$

Consequently, the calculated pathlength could be biased by the presence of the assay reagents. mPEG recovery was calculated using the absorbance of the sample (A_{Sample}), that of the positive control without serum ($A_{No\ serum}$), and that of the negative control consisting of serum without mPEG ($A_{Background}$):

$$\textit{Equation 2: Recovery}\ (\%) = (A_{Sample} - A_{Background}) \div A_{No\ serum} \times 100$$

1.9 mPEG quantification by NMR spectroscopy. Absolute mPEG concentrations were determined by NMR spectroscopy in D_2O. Prior to analysis, the water–acetonitrile mixture resulting from the serum matrix and the extraction procedure was evaporated by evaporation at

40 °C with agitation (1000 rpm) for 2 h. mPEG recovery was calculated using the mPEG methylene proton signal at 3.6 ppm from the sample (P_{Sample}), the positive control without serum ($P_{No\ serum}$), and the negative control consisting of serum without mPEG ($P_{Background}$) :

$$\textit{Equation 3: Recovery}\ (\%) = (P_{Sample} - P_{Background}) \div P_{No\ serum} \times 100$$

1.10 mPEG density. The number of surface-exposed mPEG chains $N_{mPEG,\,surface}$ was not directly measured but estimated using system-specific models. All surface density calculations rely on simplifying assumptions, including spherical geometry, uniform mPEG distribution, and constant molecular parameters. As such, the reported mPEG densities should be interpreted as comparative estimates rather than absolute values. mPEG surface density (σ; per nm^2) was defined as the number of surface-exposed mPEG chains normalized by the apparent hydrated surface area of the entity. For ease of comparison across systems, PEGylation density was expressed as the number of mPEG chains per 100 nm² (σ_{100}; per 100 nm^2):

$$\text{Equation 4}: \sigma = \frac{N_{mPEG,\,surface}}{A};\ \sigma_{100} = \sigma \times 100$$

1.10.1 mPEG density on NPs. $N_{mPEG,\,surface}$ was derived from particle volume, assumed density ($\rho_{NP} = 1.2\frac{g}{cm^3}$), the mPEG mass fraction in the formulation, and the molecular weight of mPEG.[6]

1) Average volume of a NP:

$$\text{Equation 5}: V = \frac{4}{3}\pi R_h^3$$

With,

- R_h calculated as half of the Z-average hydrodynamic diameters obtained by DLS.

2) Average mass of a NP:

$$\text{Equation 6}: m_{NP} = \rho_{NP} \cdot V$$

3) Average mass of mPEG per NP

$$\text{Equation 7}: m_{mPEG,NP} = f_{mPEG} \cdot m_{NP}$$

With,

- f_{mPEG} is the mass fraction (%) of mPEG in the nanoparticle, determined by NMR spectroscopy (Section 1.3).

4) Number of mPEG chains per NP:

$$\text{Equation 8}: N_{mPEG,total} = \frac{m_{mPEG,NP}}{M_{mPEG}} \times N_A$$

With,

- $M_{mPEG} = \frac{5000g}{mol}$
- N_A = Avogadro number, 6.023 x 10^{23} mol^{-1}

5) mPEG fraction on the surface:

$$Equation\ 9: N_{mPEG,\ surface} = N_{mPEG,total} \times \phi_{mPEG,\ external}$$

For simplicity and based on Bertrand *et al.*[3], for this calculation all mPEG chains were assumed to be solvent exposed. Accordingly, the fraction of externally exposed mPEG chains ($\phi_{mPEG,\ external}$) was set to 1. This value was not measured experimentally herein, owing to the lack of trend with this parameter on recovery.

6) Surface (hydrated):

$$Equation\ 10: A = 4\pi R_h^2$$

With,

- R_h calculated as half of the Z-average hydrodynamic diameters obtained by DLS.

7) mPEG surface density:

σ_{100} *was calculated as described above* $(Eq.4)$

1.10.2 mPEG density for the bioconjugates. $N_{mPEG,surface}$ corresponded to the experimentally determined degree of PEGylation:

1) Number of mPEG per protein:

Equation 11 : N_{mPEG} = Degree of PEGylation (measured by ^{1}H NMR)

2) Protein surface (hydrated):

A was calculated as described above (Eq.10)

With,

- R_h were taken from the biophysical literature and correspond to the native quaternary structures of the proteins in solution, including tetrameric ASNase[7], dimeric GOT and monomeric HEWL[8] and BSA[9]. The hydrodynamic radius of dimeric GOT (~3.5 nm) was estimated based on its molecular mass (~92 kDa dimer) and standard protein calibration relationships between molecular weight and hydrodynamic radius for globular proteins in solution.[10,11]

3) mPEG surface density:

σ_{100} was calculated as described above (Eq.4)

1.10.3 mPEG density for the LPs. $N_{mPEG,surface}$ was calculated from the molar fraction of lipid–mPEGs, assuming quantitative incorporation and a symmetric bilayer distribution of lipid–mPEGs, such that 50% of mPEG chains were assigned to the external leaflet. This approximation neglects potential leaflet asymmetry but is commonly used for first-order estimation of surface mPEG density. An average lipid molecular area of 0.65 nm² was assumed, consistent with reported values for phospholipids in fluid bilayers. This approximation does not account for variations induced by lipid composition or cholesterol content:

1) Particle surface (hydrated):

A was calculated as described above (Eq.10)

With,

- R_h derived from the DLS Z-average diameters.

2) Total number of lipids:

$$Equation\ 11 : N_{lipids} = \frac{A}{a_{lipids}}$$

With,

- $a_{lipids} = 0.65\ nm^2$

4) Total number of lipids-mPEG:

$$Equation\ 12 : N_{mPEG,total} = f_{mPEG,LP} \times N_{lipids}$$

With,

- $f_{mPEG,NP}$ based on the feed composition of the LP formulation.

5) Solvent-exposed fraction:

$N_{mPEG,surface}$ was calculated as described as above (Eq. 9)

For simplicity, and based on Xia *et al.*[12], mPEG chains were assumed to be equally distributed between the inner and outer surfaces of the LPs ($\phi_{mPEG,\ external} = 0.5\ (external\ leaflet)$). This value was not measured experimentally herein, owing to the lack of trend with this parameter on recovery

6) mPEG surface density:

σ_{100} was calculated as described as above (Eq.4)

1.10.4 mPEG density for the LNPs. The surface density of mPEG on LNPs was estimated using a mass-based approach, assuming spherical particles with a uniform density of 1 g/cm³ and quantitative incorporation of lipid–mPEGs. The total number of mPEG chains per nanoparticle was derived from the measured PEG mass fraction and its molar mass.

1) Average volume of an LNP, V was calculated as described above (Eq.5)

2) Average mass of an LNP:

$$\text{Equation 13}: m_{LNP} = \rho_{LNP} \cdot V$$

With,

- $\rho_{LNP} = 1.0\frac{g}{cm^3}$ (assumption based on the densities of the constituents)

3) Average mass of mPEG per LNP:

$$\text{Equation 14}: m_{mPEG,LNP} = f_{mPEG,LNP} \cdot m_{LNP}$$

With,

- $f_{mPEG,LNP}$ was calculated from each LNP initial molar feed composition and subsequently converted to mass fraction (see Lipid–$mPEG_{2kDa}$ column in Table S4).

4) Number of mPEG chains per LNP:

$$\text{Equation 15}: N_{mPEG,total} = \frac{m_{mPEG,LNP}}{M_{mPEG}} \times N_A$$

With,

- $M_{mPEG} = \frac{2000g}{mol}$
- N_A = Avogadro number, 6.022×10^{23} mol^{-1}

For simplicity, and based on Xu *et al.*[13], for this calculation ~40% $mPEG_{2kDa}$ chains were assumed to be solvent exposed ($\phi_{mPEG,\,external} = 0.4$) for the calculation of $N_{mPEG,surface}$ using equation 9. This value was not measured experimentally herein, owing to the lack of trend with this parameter on recovery.

5) mPEG density:

σ_{100} was calculated as described above (Eq.4)

All the values of mPEG density are summarized in **Table S1–4**.

1.11 Dynamic Light Scattering (DLS). Particle size distributions of PEGylated entities were measured by dynamic light scattering (DLS) using a Zetasizer Advance Lab (Malvern Panalytical) equipped with a 633 nm laser and quartz SUPRASIL® cuvettes (PCS1115, Hellma Analytics; 10 mm optical path length). Samples were prepared at an initial concentration of approximately 1 mg mL^{-1}, with a final volume of 1 mL, and were analyzed without filtration to preserve sample integrity. Measurements were performed at 25 °C either in water (refractive index 1.33, viscosity 0.887 mPa s) or in a 75% acetonitrile / 25% water (*v*/*v*) mixture (refractive index 1.34, viscosity 0.54 mPa s). Each sample was measured in triplicate. Only datasets meeting quality control criteria were considered, defined as a mean count rate between 100 and 500 kcps and an attenuator setting between 1 and 10 (instrument limit: 11).

1.12 Preparation of rat liver lysate. Tissues from male transgenic Sprague-Dawley rats expressing germ cell-specific EGFP (GCS-EGFP) were recovered from a pre-existing biobank in accordance with institutional ethical guidelines (CIPA 2504-05) and were thawed, weighed, and homogenized at room temperature in 4 mL of PBS containing 0.5% (*v*/*v*) Triton X-100 in 15 mL polypropylene tubes. Homogenization was performed using a probe homogenizer (75% power) for three 60 s cycles with 30 s intervals between cycles. No protease inhibitors were included.

1.13 Optical microscopy. Optical microscopy was employed to qualitatively assess aggregate and precipitate formation following deproteinization. Samples were prepared by mixing PEGylated entities (1 mg mL^{-1}) in 10 µL of human serum. Three volume equivalents of deproteinization solvent were added, followed by vortex mixing. For acidic pre-treatment experiments, samples were first incubated with one volume equivalent of HCl at 37 °C for 1 h prior to the addition of three volume equivalents of deproteinization solvent and vortex mixing. Subsequently, 10 µL aliquots were placed onto standard glass microscope slides and covered with glass coverslips. Samples were examined at room temperature by bright-field optical microscopy using a Motic AE2000 binocular microscope at a final magnification of 40×. Representative images were acquired immediately following sample preparation, with one representative image shown per sample.

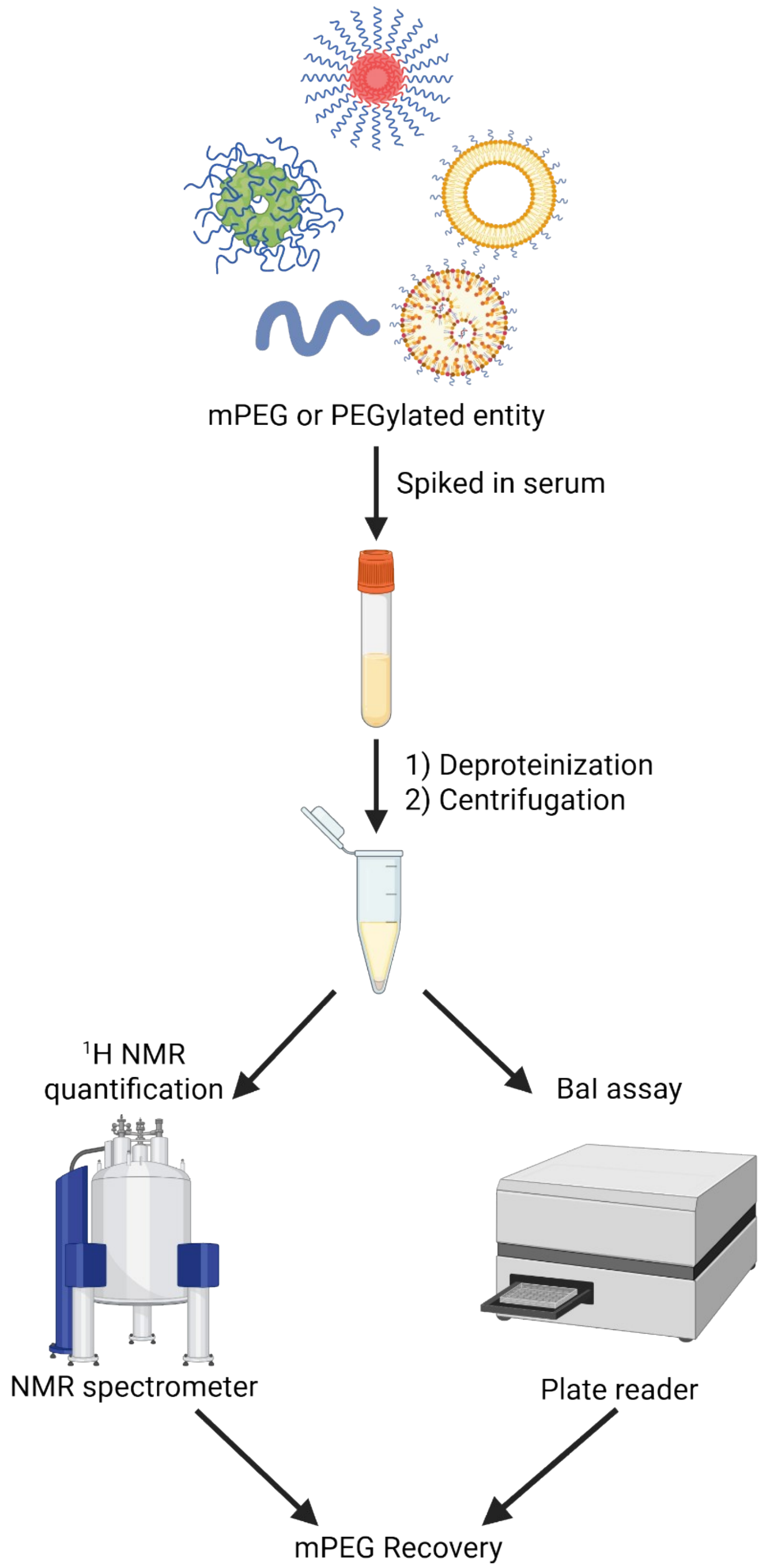

Scheme S1 | Experimental workflow for serum deproteinization and mPEG recovery analysis. mPEG or PEGylated entities were spiked in serum and subjected to deproteinization followed by centrifugation. mPEG recovery was quantified by BaI assay by absorbance at 535 nm or ^{1}H NMR spectroscopy.

Table S1 | Physicochemical characteristics of protein–mPEG bioconjugates.

Bioconjugate	Protein	MW_{mPEG} (kDa)	Feed mPEG: NH_2 ratio	Degree of PEGylation (NMR)	$M_{n,SEC}$ (kDa)	Đ	R_h (nm)	σ_{100} (mPEG per 100 nm^2)
1	ASNase	0.55	5:1	11	142	1.1	4.3	4.7
2		2		2.0	108	1.2	4.3	0.9
3*				7.8	107	1.3	4.3	3.4
4		5		5.5	97	1.2	4.3	2.4
5	HEWL			8.7	20	1.1	1.9	19.2
6	GOT			28	119	1.2	3.5	18.2
7	BSA			33	107	1.3	3.4	22.7
8†	ASNase		N/A	19	345	1.1	4.3	8.3
9		20	5:1	5.5	138	1.1	4.3	2.4

*Conjugate prepared with $mPEG_{2kDa}$–aldehyde.

†Commercial formulation of Oncaspar® (pegaspargase) by Servier.

Note: mPEG surface density (σ_{100}) was calculated as described in Section 1.10.2

Table S2 | Composition and key properties of PLA/PLGA–mPEG nanoparticles.

NP Characteristics		Polyester-based polymers			Characteristics			
		$PLGA_{30kDa}$ (mol%)	$PLGA_{95kDa}$ (mol%)	PLA_{25kDa}–$mPEG_{5kDa}$ (mol%)	R_h (nm)	Đ	mPEG content by NMR (wt%)	σ_{100} (mPEG per 100 nm^2)
A	Common composition	10	0	90	49	1.2	13.5	32
B	Less mPEG	70	0	30	58	1.1	4.6	13
C	Larger size	20	0	80	64	1.2	12.1	37
D	PLGA core, Less mPEG	0	60	40	90	1.1	6.1	26

Note: mPEG surface density was calculated as described in Section 1.10.1, and total mPEG content was measured as described in Section 1.10.1.

Table S3 | Lipid composition and physicochemical properties of liposomes.

LPs		Zwitterionic phospholipids		Sterol	Lipids–mPEG$_{2kDa}$		Characteristics				
		DSPC (mol%)	DOPE (mol%)	Chol. (mol%)	DMG (mol%)	DSPE (mol%)	R_h (nm)	Đ	[PO_4^{3-}] (mM)	[Chol.] (mM)	σ_{100} (mPEG per 100 nm^2)
A	Ref LP[1]	56.5	0	38.5	0	5	58	1.1	5.82	2.64	3.8
B	No mPEG	60	0	40	0	0	170	1.4	1.86	0.61	0
C	Less mPEG	60	0	38.5	0	1.5	59	1.1	5.85	1.65	1.2
D	DOPE	0	56.5	38.5	0	5	65	1.1	3.76	2.06	3.8
E	DMG	56.5	0	38.5	5	0	58	1.1	4.39	2.58	3.8

[1]The formulation is similar to Doxil® and Caelyx®.

Note: mPEG surface density was calculated as described in Section 1.10.3

Table S4 | Lipid composition and physicochemical properties of lipid nanoparticles.

LNPs		Zwitterionic phospholipids		Sterol	Ionizable cationic lipids		Lipid–mPEG$_{2kDa}$	Characteristics			
		DSPC (mol%)	DOPE (mol%)	Chol. (mol%)	DODAP (mol%)	MC3 (mol%)	DMG–mPEG$_{2kDa}$ in mol% ($f_{mPEG,LNP}$)	R_h (nm)	Đ	[Chol.] (mg/mL)	σ_{100} (mPEG per 100 nm^2)
A	Ref LNP[1]	10	0	38.5	0	50	1.5 (0.066)	86	1.2	0.37	23
B	Contains nucleic acid[2]	10	0	38.5	50	0	1.5 (0.063)	79	1.1	0.57	20
C	More mPEG	10	0	37	0	50	3 (0.124)	85	1.1	0.47	42
D	DOPE	0	10	38.5	0	50	1.5 (0.066)	83	1.1	0.32	22
E	DODAP	10	0	38.5	50	0	1.5 (0.063)	74	1.1	0.83	19

[1]The formulation is similar to Onpattro®.

[2]poly(A) was encapsulated in LNPs via rapid mixing of ethanolic and aqueous phases using a T-junction. Detailed conditions are described in Section 1.5.

Note: mPEG surface density was calculated as described in Section 1.10.4

Table S5 | Residual signals in the supernatant following deproteinization under different conditions versus those in full serum. Data are presented as Mean ± s.d. (n = 1 in triplicate).

PEGylated entities	Extraction solvent	Absorbance at 280 nm (% vs. serum)	Tryptophan fluorescence (% vs. serum)
H_2O	ACN	0.8 ± 0.3	0.35 ± 0.03
	ACN*	0.7 ± 0.1	0.20 ± 0.04
	EtOH	1.2 ± 0.5	1.3 ± 0.4
	EtOH*	1.3 ± 0.2	1.1 ± 0.1
	Acetone	N/A	3.7 ± 0.4
	Acetone*	N/A	0.054 ± 0.006
$mPEG_{5kDa}$	ACN	0.24 ± 0.02	0.107 ± 0.007
	ACN*	0.4 ± 0.2	0.053 ± 0.005
	EtOH	0.6 ± 0.1	0.058 ± 0.009
	EtOH*	0.5 ± 0.7	0.370 ± 0.009
	Acetone	N/A	0.2 ± 0.1
	Acetone*	N/A	0.400 ± 0.006
ASNase–$mPEG_{5kDa}$	ACN	0.38 ± 0.07	0.008 ± 0.005
	ACN*	0.31 ± 0.05	0.270 ± 0.005
	EtOH	0.7 ± 0.2	11.1 ± 0.3
	EtOH*	0.8 ± 0.2	0.55 ± 0.05
	Acetone	N/A	0.36 ± 0.02
	Acetone*	N/A	0.141 ± 0.008
NP_A	ACN	0.29 ± 0.07	0.96 ± 0.02
	ACN*	0.24 ± 0.02	0.29 ± 0.02
	EtOH	0.9 ± 0.5	2.55 ± 0.02
	EtOH*	1.0 ± 0.5	0.47 ± 0.09
	Acetone	N/A	0.79 ± 0.02
	Acetone*	N/A	0.322 ± 0.001
LP_A	ACN	0.29 ± 0.02	0.183 ± 0.002
	ACN*	0.19 ± 0.02	0.360 ± 0.005
	EtOH	0.9 ± 0.1	0.083 ± 0.002
	EtOH*	1.2 ± 0.7	0.187 ± 0.009
	Acetone	N/A	0.03 ± 0.01
	Acetone*	N/A	0.08 ± 0.02
LNP_A	ACN	0.31 ± 0.05	0.349 ± 0.005
	ACN*	0.40 ± 0.07	0.564 ± 0.009
	EtOH	1.4 ± 0.2	0.449 ± 0.002
	EtOH*	1.8 ± 0.5	0.031 ± 0.007
	Acetone	N/A	0.238 ± 0.001
	Acetone*	N/A	0.094 ± 0.009

Asterisks (*) denote acid-pretreated samples. Note: Values for acetone are reported as not available "N/A" due to its strong absorbance at 280 nm, which prevents accurate protein quantification.

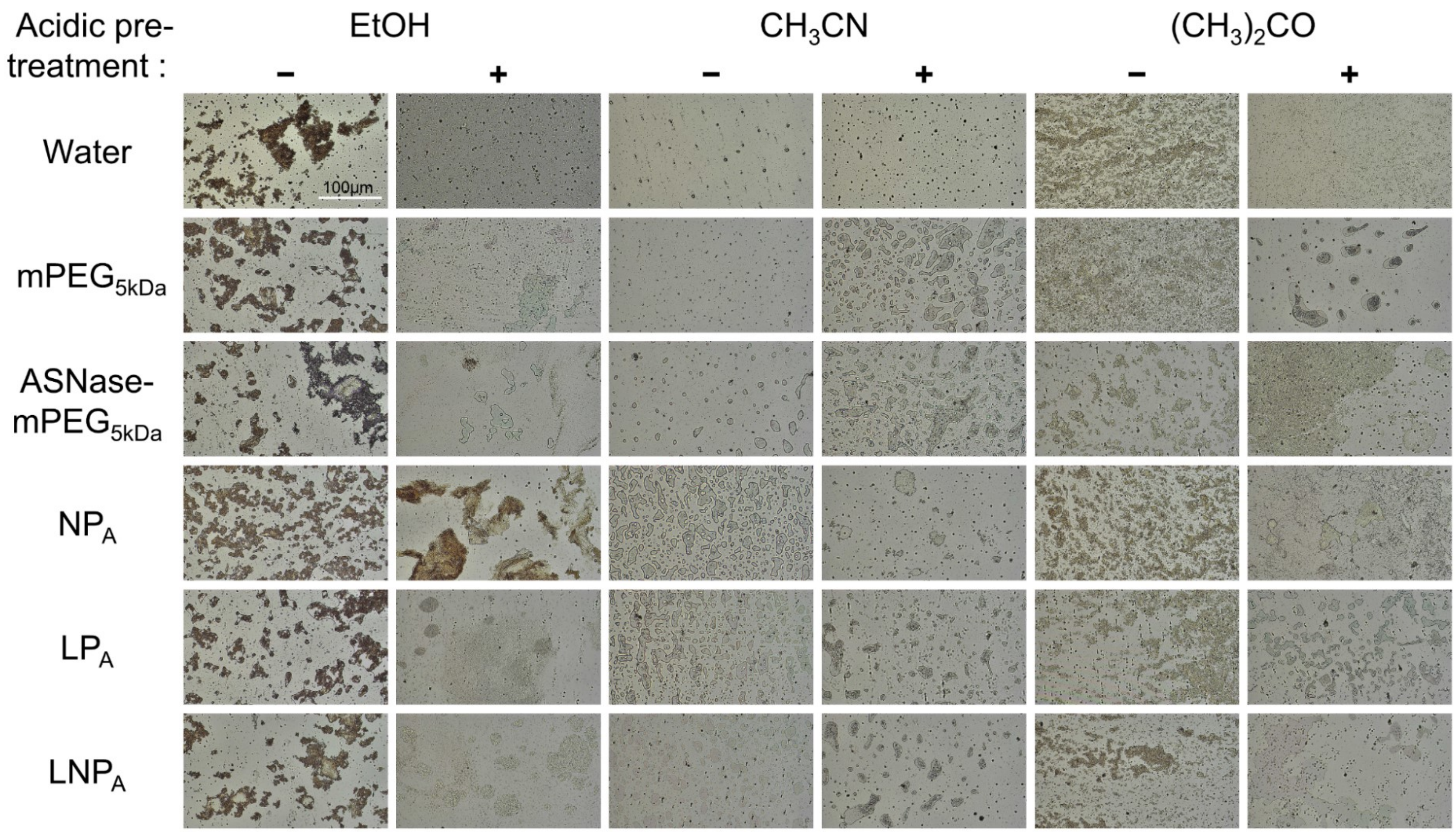

Figure S1 | Representative optical microscopy images of serum precipitates following deproteinization with ethanol, acetonitrile, or acetone. Ethanol produced the largest precipitates, acetonitrile the smallest, and acetone an intermediate size. PEGylated entities did not visibly alter precipitate appearance. Acidic pre-treatment (500 mM HCl, 1 h at 37 °C) generally led to more dispersed precipitates during ethanol precipitation. Images were acquired immediately after preparation using bright-field optical microscopy (40× magnification).

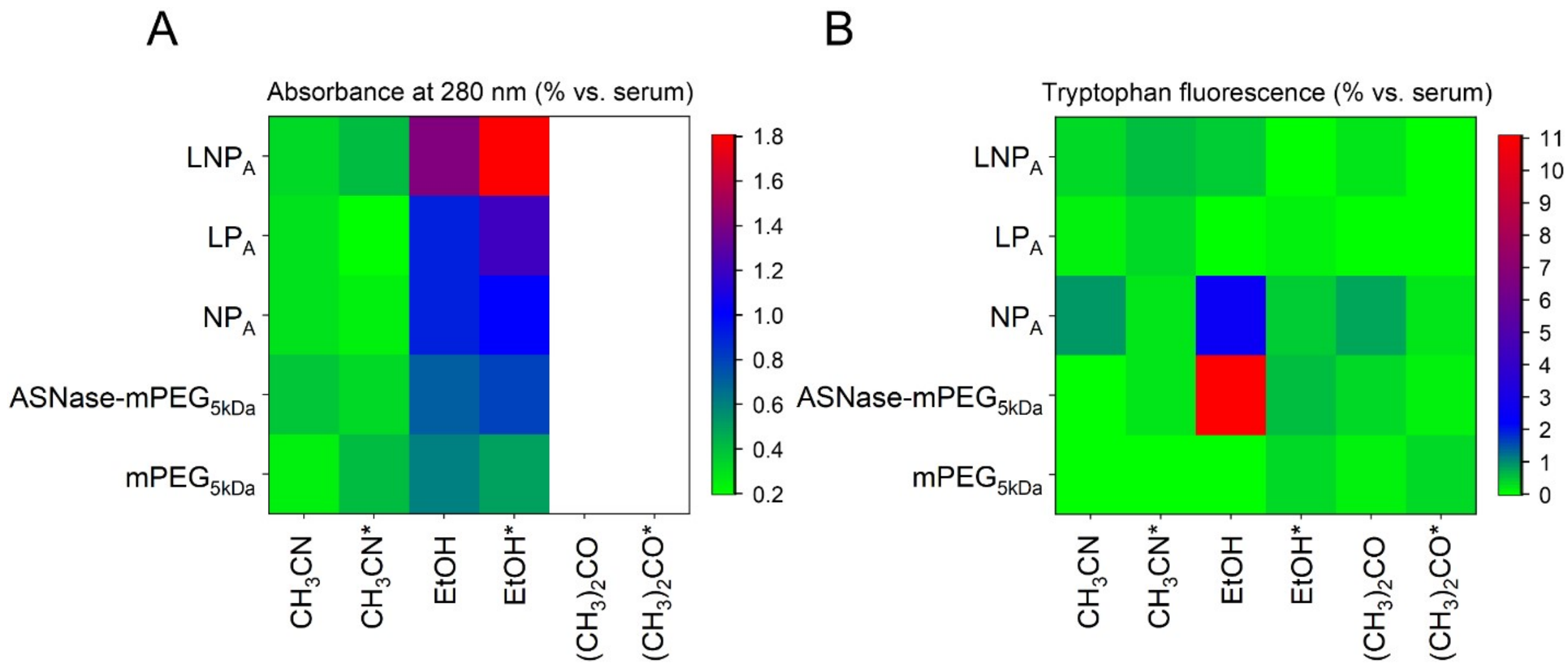

Figure S2 | Residual matrix signals in the supernatant following deproteinization. (A) Residual absorbance at 280 nm after deproteinization. **(B)** Residual tryptophan fluorescence after deproteinization. Values for acetone are reported as "N/A" due to its strong absorbance at 280 nm and are displayed in white in the heatmaps. Corresponding numerical values are provided in **Table S5**. Asterisks (*) denote acidic pretreated samples.

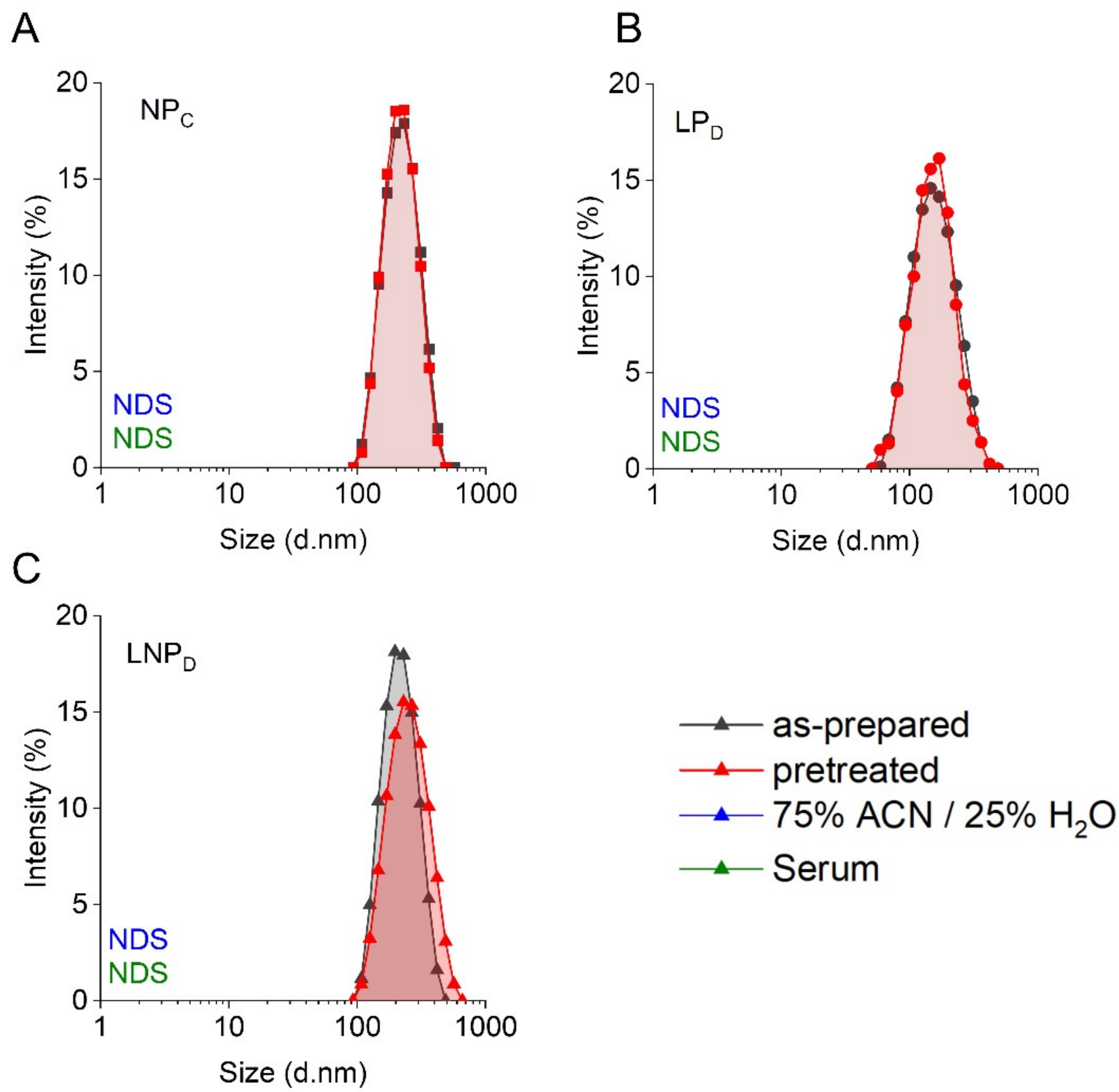

Figure S3 | Dynamic light scattering (DLS) analysis of NP$_C$, LP$_D$, and LNP$_D$. Size distributions were measured under the following conditions: as-prepared in 10 mM HEPES-buffered saline, pH 7.4, after acid pretreatment (1 h incubation at 37 °C in 500 mM HCl), in 75% acetonitrile / 25% water (v/v), or after incubation in human serum followed by acetonitrile-based deproteinization. Data for as-prepared and pre-treated samples generally has a mean count rate between 150–350 kcps with an attenuator setting between 5–7. The conditions identified as “NDS” were considered to have no detectable signal because low count rates were observed even when the attenuator value was ≥10 (maximum of 11). Data were acquired from a single preparation (n = 1, in triplicate).

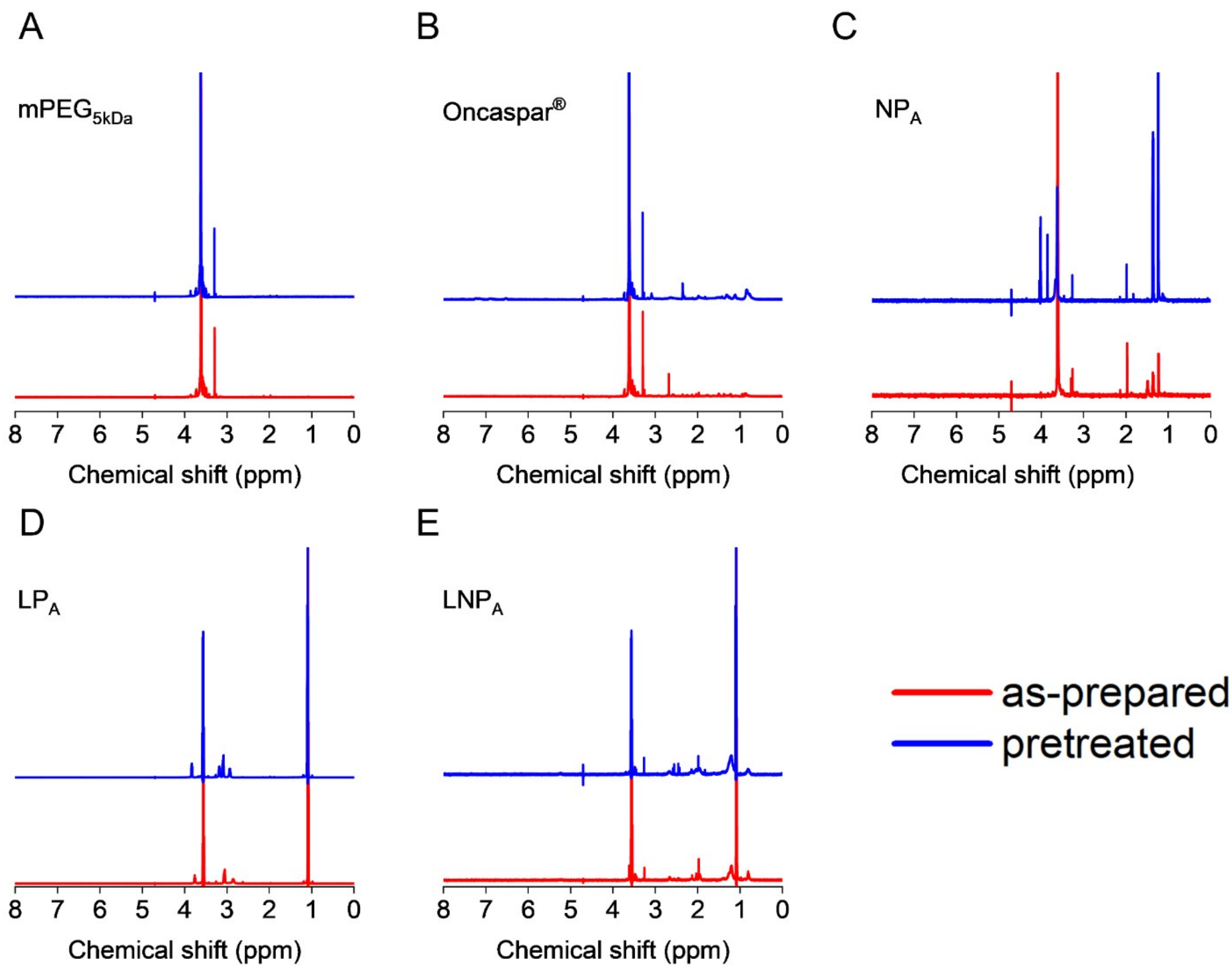

Figure S4 | Representative ^{1}H NMR spectra of PEGylated entities as-prepared and after acidic pre-treatment. (A) $mPEG_{5kDa}$, (B) PEGylated asparaginase (Oncaspar®), (C) mPEG-PLA/PLGA nanoparticles (NP_A), (D) liposome A (LP_A), and (E) lipid nanoparticle A (LNP_A). Acidic pre-treatment (500 mM HCl, 1 h at 37 °C) did not result in any obvious changes, except for NP_A, which showed evidence of partial ester hydrolysis. Spectra were acquired in D_2O.

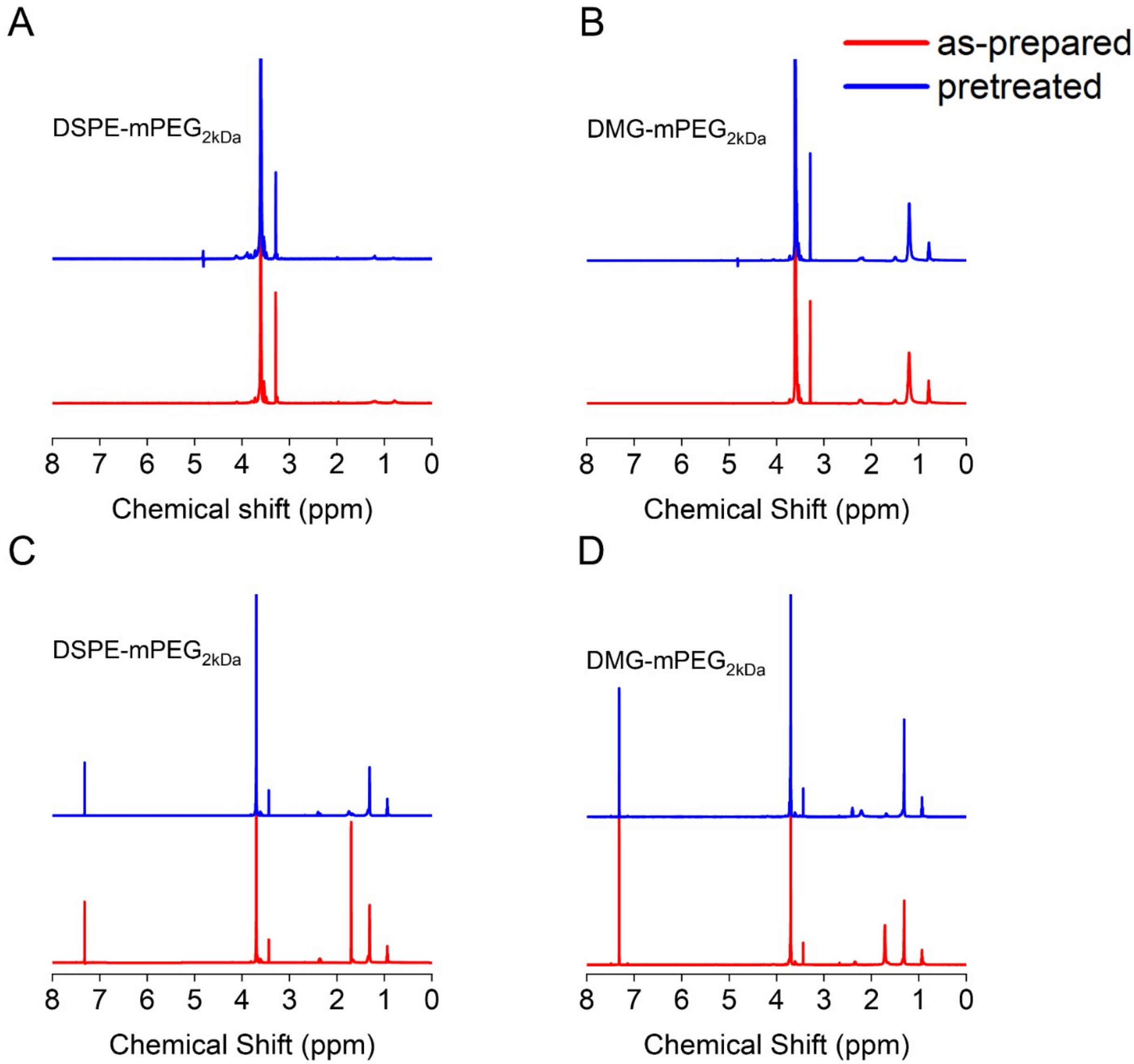

Figure S5 | Representative ^{1}H NMR spectra of DSPE-mPEG$_{2kDa}$ and DMG-mPEG$_{2kDa}$ before (as-prepared) and after acidic pre-treatment. Spectra were acquired in D_2O for DSPE-mPEG$_{2kDa}$ (A) and DMG-mPEG$_{2kDa}$ (B), and in $CDCl_3$ for DSPE-mPEG$_{2kDa}$ (C) and DMG-mPEG$_{2kDa}$ (D). For C and D, aqueous samples were lyophilized and reconstituted in $CDCl_3$. The broadened resonance around 1.8 ppm was only observed after acidic pre-treatment when the sample was lyophilized and reconstituted in $CDCl_3$. The corresponding spectrum acquired directly in D2O did not show this feature. Importantly, the characteristic resonances of the PEG and lipid moieties remained unchanged in both solvents, supporting the conclusion that the acidic pre-treatment did not measurably alter the chemical structure.

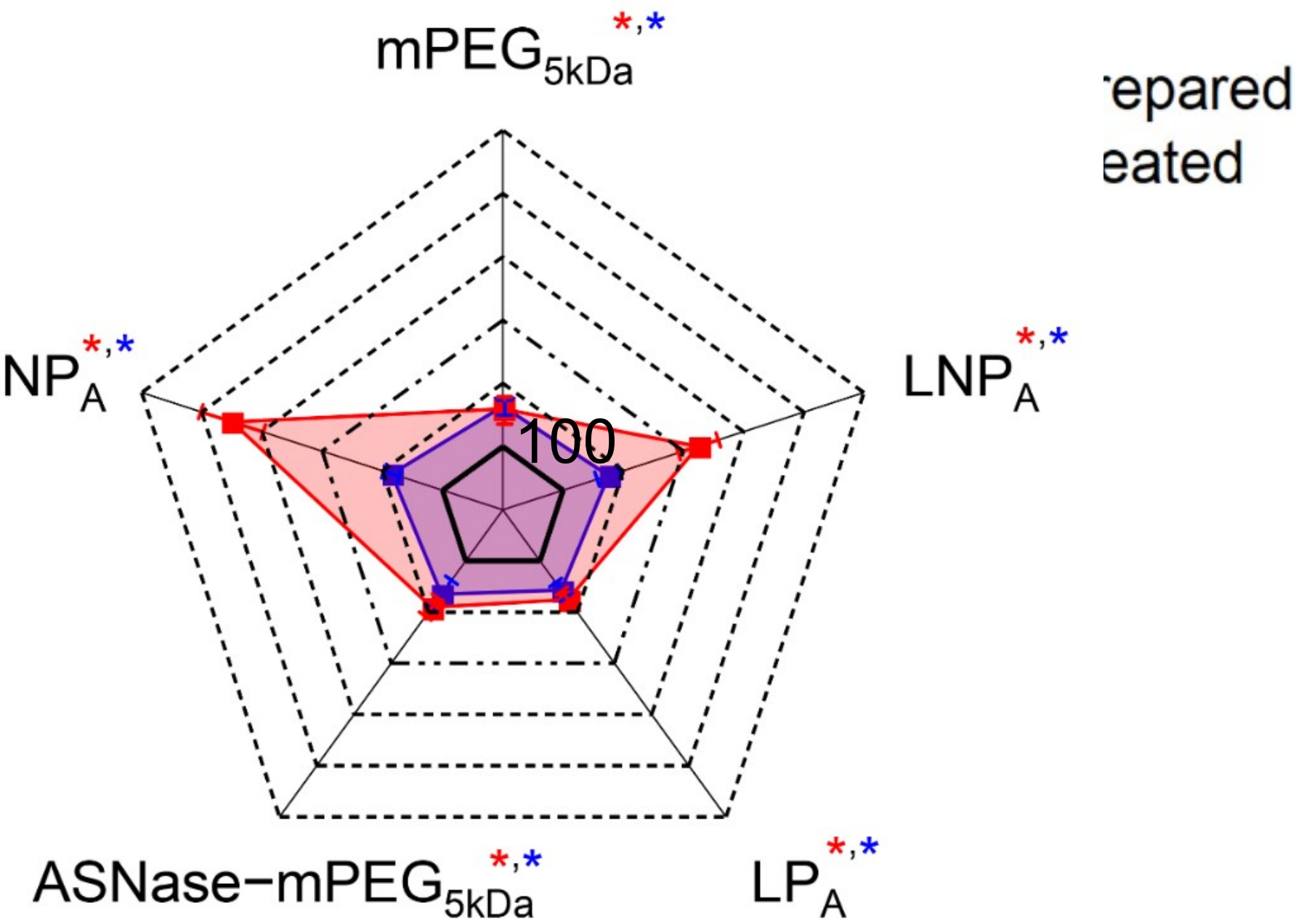

Figure S6 | Recovery of PEGylated entities following prolonged exposure to liver lysate was evaluated by the BaI assay. Apparent recovery values for all systems remained well above the expected 100% (bold line), both with and without acidic pre-treatment, throughout the incubation period, precluding quantitative interpretation. After 24 h of incubation, mPEG was detectable following acetonitrile extraction, with or without acidic pre-treatment to dissociate adsorbed proteins. Recovery values exceeded 100%, especially for NP_A and LNP_A, likely reflecting protein corona formation as seen in serum. Acidic pre-treatment narrowed the distribution to ~200%, with no significant differences between nanosystems over time. Liver lysate control samples (serum + acetonitrile, data not shown) contained substantial background molecules, partially removed by acid digestion.

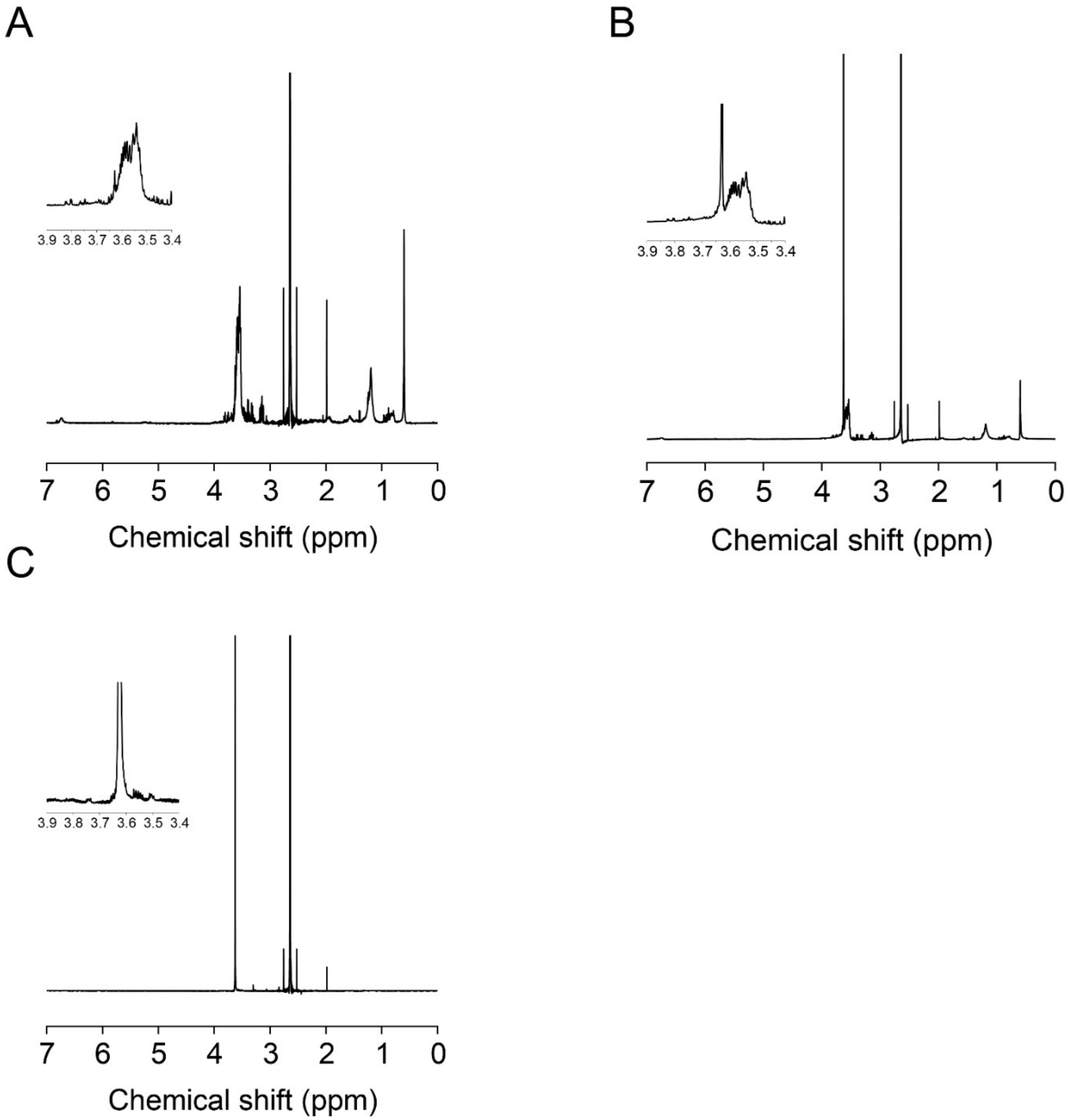

Figure S7 | ^{1}H NMR spectra of $mPEG_{5kDa}$ in rat hepatic lysate. (A) Hepatic lysate alone, **(B)** hepatic lysate spiked with $mPEG_{5kDa}$, and **(C)** $mPEG_{5kDa}$ dissolved in water. Spectra were normalized using a spiked DMSO internal standard. The characteristic PEG $–CH_2CH_2O–$ resonance at ~3.64 ppm is clearly observed in water but is strongly attenuated and overlaps with endogenous signals in hepatic lysate, highlighting the limitations of direct NMR spectroscopy detection of mPEG in complex biological matrices. Insets show an expanded view of the 3.4–3.9 ppm region.

Supplementary References

S1 K. Coutu et al., *Biomacromolecules*, 2025, 26, 3689–3699.

S2 A. Zaghmi et al., *Data Brief*, 2019, 25, 104037.

S3 N. Bertrand et al., *Nat. Commun.*, 2017, 8, 777.

S4 L. C. Paweletz, N. Labedzki and T. Günther Pomorski, *J. Liposome Res.*, 2025, 35, 86–93.

S5 Y. H. Itoh, T. Itoh and H. Kaneko, *Anal. Biochem.*, 1986, 154, 200–204.

S6 G. P. Meneguetti et al., *PLoS One*, 2019, 14, e0211951.

S7 A. S. Parmar and M. Muschol, *Biophys. J.*, 2009, 97, 590–598.

S8 A. Valstar, M. Almgren, W. Brown and M. Vasilescu, *Langmuir*, 2000, 16, 922–927.

S9 C. R. Cantor and P. R. Schimmel, *Biophysical Chemistry*, W. H. Freeman, 1980.

S10 B. J. Berne, R. Pecora and P. Dover, *Dynamic Light Scattering: With Applications to Chemistry, Biology and Physics*, Dover Publications, 2018.

S11 Y. Xia, J. Tian and X. Chen, *Biomaterials*, 2016, 79, 56–68.

S12 Q. Xu et al., *J. Control. Release*, 2013, 170, 279–286.

S13 K. Kovshova et al., *Biomolecules*, 2024, 14, 1601.
